# A Versatile in Vivo DNA Assembly Toolbox for Fungal Strain Engineering

**DOI:** 10.1021/acssynbio.2c00159

**Published:** 2022-09-20

**Authors:** Zofia Dorota Jarczynska, Katherina Garcia Vanegas, Marcus Deichmann, Christina Nørskov Jensen, Marouschka Jasmijn Scheeper, Malgorzata Ewa Futyma, Tomas Strucko, Fabiano Jares Contesini, Tue Sparholt Jørgensen, Jakob Blæsbjerg Hoof, Uffe Hasbro Mortensen

**Affiliations:** † Eukaryotic Molecular Cell Biology, Section for Synthetic Biology, Department of Biotechnology and Biomedicine, Technical University of Denmark, 2800 Kongens Lyngby, Denmark; ‡ The Novo Nordisk Foundation Center for Biosustainability, Technical University of Denmark, 2800 Kongens Lyngby, Denmark

**Keywords:** filamentous fungi, in vivo DNA assembly, CRISPR, gene targeting, gene expression

## Abstract

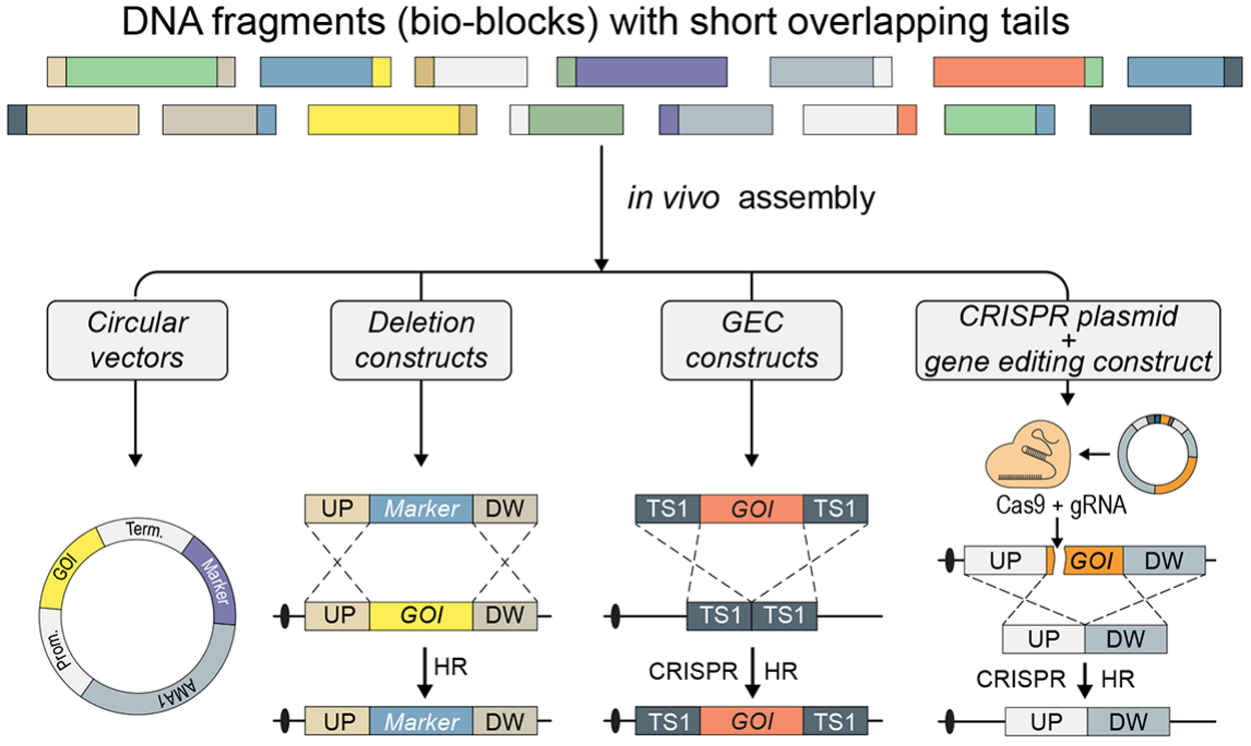

Efficient homologous recombination in baker’s yeast allows accurate fusion of DNA fragments via short identical sequence tags in vivo. Eliminating the need for an *Escherichia coli* cloning step speeds up genetic engineering of this yeast and sets the stage for large high-throughput projects depending on DNA construction. With the aim of developing similar tools for filamentous fungi, we first set out to determine the genetic- and sequence-length requirements needed for efficient fusion reactions, and demonstrated that in nonhomologous end-joining deficient strains of *Aspergillus nidulans*, efficient fusions can be achieved by 25 bp sequence overlaps. Based on these results, we developed a novel fungal in vivo DNA assembly toolbox for simple and flexible genetic engineering of filamentous fungi. Specifically, we have used this method for construction of AMA1-based vectors, complex gene-targeting substrates for gene deletion and gene insertion, and for marker-free CRISPR based gene editing. All reactions were done via single-step transformations involving fusions of up to six different DNA fragments. Moreover, we show that it can be applied in four different species of Aspergilli. We therefore envision that in vivo DNA assembly can be advantageously used for many more purposes and will develop into a popular tool for fungal genetic engineering.

## Introduction

Filamentous fungi play a crucial role in the ecosystem as they facilitate decomposition and recycling of organic matter and nutrients.^1−3^ Due to their saprophytic, and sometimes symbiotic or pathogenic, lifestyles they produce a multitude of different enzymes that can be used to degrade matter, and secondary metabolites that can be used in communication or act as toxins.^4,5^ Some of these are already important products of the food, biotech, and pharma industries, but the vast majority remains to be discovered.^6^ Hence, there is a strong desire to accelerate basic, applied, agricultural, and medical fungal research, and efficient genetic-engineering tools that can be used as the basis for performing high-throughput genetic-engineering experiments are in demand.^7^ Over the years, the fungal genetic-engineering toolbox has been ever expanding and includes, e.g., episomal AMA1 based plasmids and circular mini chromosomes, collections of synthetic biology based building blocks including markers, promoters, and terminators, etc., and efficient gene-targeting and gene-editing technologies based on strains that are deficient in nonhomologous end-joining (NHEJ) and/or by CRISPR technologies. The majority of the tools require the assembly of DNA constructs using *Escherichia coli* based cloning strategies or in vitro assembly by fusion-PCR reactions.^8^ For high-throughput experiments, these DNA assembly processes are often bottlenecks in strain construction due to the time requirements, costs, low efficiency of multipart assembly, and need of optimization.

In the yeast *Saccharomyces cerevisiae*, DNA assembly can be efficiently performed in vivo by exploiting that PCR fragments containing short homologous sequence overlaps, typically around 30–50 bps, can be accurately fused by homologous recombination (HR). This methodology was pioneered by the observation that a single DNA fragment could be efficiently inserted into a linearized plasmid by HR in yeast.^9^ Using the same approach, it was later demonstrated that several fragments could be fused by HR in vivo to create more complex plasmids,^10^ or to establish multipart gene-targeting substrates containing entire pathways.^11,12^ DNA assembly in *S. cerevisiae* has even been used to drive research in filamentous fungi by using it as a host for DNA assembly, e.g., allowing for the construction of the fungal AMA1 based mini chromosomes.^13^

In Aspergilli, PCR fragments have also been puzzled together in vivo by HR; e.g., in split-marker based experiments or to assemble gene clusters.^14−18^ However, in these cases, fusions were typically mediated by long 500–1500 bp homology sequences present in the ends of the fragments, which are too long to be added to a new sequence via a primer tail. Nonpriming tails should ideally be as short as possible, typically 20–60 nucleotides, to be cost efficient and to reduce the chance of influencing priming efficiency due to increased risks of intraprimer secondary structures, primer dimer formation, and lowered primer quality. To fully exploit in vivo recombination in fungal genetic engineering, including high-throughput experiments, inexpensive, simple, and robust methods for filamentous fungi allowing PCR fragments to be orderly stitched together are necessary. However, this task may be complicated by the fact that efficient gene targeting in filamentous fungi, even in NHEJ-deficient strains, requires longer stretches of DNA homology sequences, typically 1–2 kb (500 bp in NHEJ deficient strains),^19−22^ as compared to the much shorter sequences, down to 30–100 bp,^23^ which is required by *S. cerevisiae*. However, when gene targeting is stimulated by the presence of DNA double strand break (DSB) in the target sequence, albeit in a chromosome or in an AMA1 vector, a linear DNA fragment can be inserted by short 60 bp homology sequences in *Penicillium*.^24^ Similarly, 90 base single-stranded oligonucleotides can be used for directed chromosomal mutagenesis in species of Aspergilli, as they are used as templates to repair DNA DSBs indicating that short sequences can be used for HR mediated repair.^25,26^ In this report, we have expanded on these observations and investigated the impact of homology length on HR directed DNA fragment assembly. Moreover, based on the results, we have developed a versatile fungal DNA assembly toolbox, which offers simple and efficient plasmid- and gene-targeting substrate construction in vivo via HR mediated multifragment assembly, and demonstrated its applicability in four different filamentous fungi: *A. aculeatus*, *A. nidulans*, *A. niger*, and *A. oryzae*.

## Results

### Assembly of DNA Fragments in Vivo by HR via Short Sequence Tags Requires Elimination of the NHEJ DNA Repair Pathway

**Figure 1:**
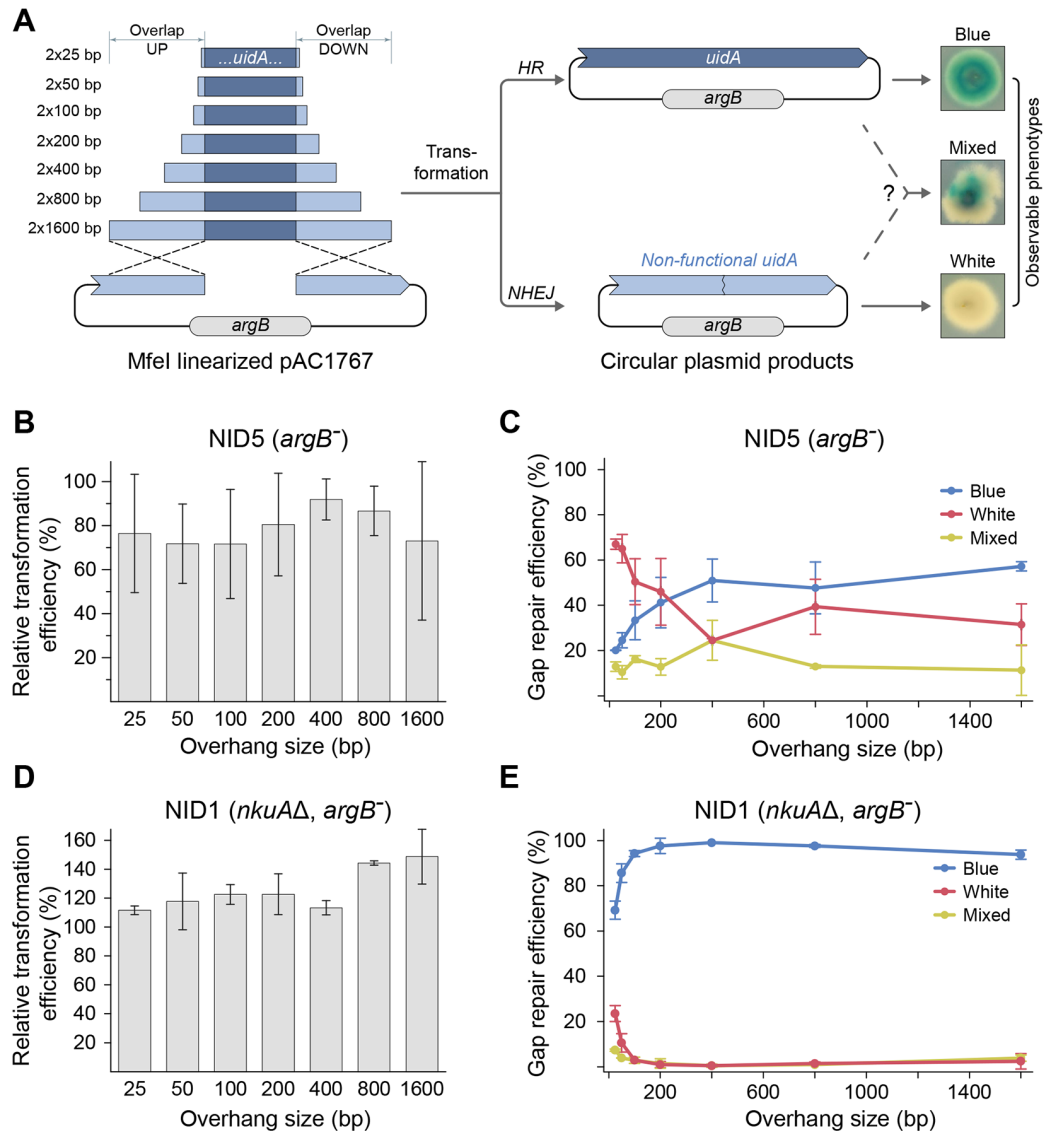
Short sequences are sufficient to mediate efficient homology-directed end-joining of DNA fragments. (A) Gap repair assay to determine how the lengths of the sequence overlaps influence the efficiencies of homology-directed end-joining fusions; see text for details. Cotransformation with linearized pAC1767 plasmid and a *uidA* repair fragment sets the stage for gap repair using *argB* as a selectable marker. Repair mediated by HR results in blue colonies as *uidA* functionality is restored. Repair by NHEJ, or by flawed HR, results in white colonies. Colonies containing blue-white sectors most likely represent heterokaryons where individual nuclei may contain a plasmid formed either by HR or by NHEJ. (B,C) Cotransformation of NID5 with linearized pAC1767 and *uidA* repair fragments containing different length of *uidA* sequence overlaps as indicated. In (B), cotransformation efficiency is shown relative, in percent, to the efficiency obtained with the circular reference plasmid pAC1688. In (C), numbers of blue, white, and blue-white colonies obtained in the individual cotransformation experiments as indicated. (D,E) Same as (B) and (C), respectively, except that cotransformations were performed with NID1.

Development of methods, which are based on orderly DNA fragment assembly via HR, requires insights into the DNA fragment fusion process. We therefore developed a gap repair assay that measures the efficiency of DNA HR mediated end-fusions via a blue/white colony readout based on *E. coliuidA*, which encodes a β-glucuronidase.^27^ The assay uses an *argB-*AMA1 plasmid (pAC1767; see Supplementary Table S1) containing a nonfunctional allele of the *uidA* marker gene, *uidA*-5tr (see Supplementary Figure S1). Specifically, *uidA*-5tr is generated by deleting a MfeI fragment of *uidA* that contains 567 bp of promoter sequence, the USER cassette (14 bp), and 130 bp of coding sequence in a process that leads to formation of a single MfeI cut site. Importantly, the MfeI site in pAC1767 is unique, and MfeI enzyme can therefore be used to linearize the plasmid by introducing a DNA DSB in *uidA*-5tr in vitro to set the stage for gap repair experiments; see Figure 1A.

In the DNA assembly assay, a fungus is cotransformed with MfeI-linearized pAC1767 in the absence or presence of a linear *uidA* repair fragment. The repair fragment contains the missing *uidA* sequence as well as *uidA* sequences up- and downstream of the MfeI cleavage site. The latter sequences overlap with the ends of MfeI-linearized pAC1767 to allow for HR-mediated gap repair of pAC1767. Three classes of transformants may be generated in the assay, Figure 1A. The first class contains blue transformants and represents desirable events where the repair fragment is used to restore the *uidA* marker by HR. The second class contains white transformants and represents undesirable events where the *uidA* marker is not functionally restored. We envision that the most likely events causing this phenotype are (i) circularization of pAC1767 by NHEJ, (ii) integration of pAC1767 (or a part of its sequence information including *argB*) into the genome by NHEJ or by HR, and (iii) class one events where *uidA* functionality is compromised by, e.g., DNA polymerase errors during repair. The third class is composed by colonies that display white and blue sectors. Such transformants likely represent heterokaryons containing a mix of the events described for class one and class two transformants.

We then applied the assay to assess whether the length of sequence overlaps influences the efficiency of HR mediated gap repair. Specifically, we created a set of linear *uidA* repair fragments containing varying lengths (25–1600 bps) of flanking *uidA* homology sequences. Each fragment in the set contained the same length of flanking *uidA* homology sequence in each end; see Figure 1A. Accordingly, an *A. nidulans argB*^*–*^ strain (NID5; see Supplementary Table S2) was cotransformed with linearized pAC1767 and *uidA* repair fragments. In parallel, and in order to normalize the number of transformants obtained in individual experiments, we also transformed *A. nidulans* with the circular plasmid pAC1688 (see Supplementary Table S1), which is identical with pAC1767 except that it encodes a functional *uidA* marker, Figure 1A. In this way, we could calculate relative transformation efficiencies for the different cotransformation experiments to allow for comparisons. In all cotransformation experiments, transformants were easily generated and the relative transformation efficiencies did not appear to depend on the length of the homologous overlaps between *uidA* repair fragments and linearized pAC1767. Hence, the lowest (obtained with 100 bps overhangs) and the highest transformation efficiency (obtained with 400 bps overhangs) only varied 1.3-fold and the difference was not significant (*p*-value ≥ 0.44); see Figure 1B. We then examined whether the transformants contained functional *uidA* or not. Accordingly, transformants obtained from the different experiments were transferred to solid MM + X-Gluc plates and analyzed for functional *uidA* activity, Figure 1C. For experiments producing 20–99 transformants or more than 100 transformants, all or at least 100 of the transformants, respectively, were tested in this manner. For experiments generated from DNA fragments containing long homologous sequence overlaps, 400–1600 bps, blue colonies were easily obtained. In fact, 50–60% of the colonies were entirely blue, 10–20% were white, and 20–40% were of mixed color. These results indicate that HR is the preferred pathway with this range of fragments. However, as the sequence overlaps were shortened, this preference disappeared and NHEJ (or flawed HR) became the dominating plasmid rescue pathway. With *uidA* sequence overlaps 25 and 50 bp long, only 20 and 25% of the colonies were entirely blue, respectively. Accordingly, efficient in vivo assembly of DNA fragments by HR in strains that also contain the competing NHEJ pathway requires ≥400 bps of sequence identity between the fragments to be merged.

Next, we assessed the influence of the NHEJ pathway on the efficiency of HR mediated gap repair. Hence, we performed the same experiment with the *A. nidulans* strain NID1 (see Supplementary Table S2), which in addition to *argB*^*–*^ contains a deletion of *nkuA* that compromises the NHEJ DNA repair pathway.^20^ Like with NID5, the relative transformation efficiencies did not appear to be dramatically influenced by the sizes of the homologous sequence overlaps, Figure 1D. In this set of experiments, the lowest (obtained with 25 bps overhangs) and the highest numbers (obtained with 1600 bps overhangs) varied 1.3-fold, and this difference was also not significant (*p*-value ≥ 0.21). Importantly, and unlike with NID5, assessment of color on solid X-Gluc medium showed that the vast majority of colonies, ≥90%, obtained with NID1 were entirely blue when the overlapping *uidA* sequences were between 100 bps and 1600 bps, Figure 1E. Even with overlapping sequences of 25 bps and 50 bps we observed that 70% and 85% of the transformants were entirely blue, respectively. These gains in blue colony numbers are due to reductions in the formation of both white colonies and colonies of mixed color, strongly indicating that the majority of white colonies obtained with NID5 are due to repair by NHEJ. The fact that the dramatic reductions in the numbers of white colonies were not accompanied by equivalent reductions in the transformation efficiency strongly indicates that linear vector fragments, which would normally be repaired by NHEJ, are efficiently channeled into the HR repair pathway rather than being lost. Lastly, we investigated the fidelity of in vivo DNA fusions by sequencing PCR fragments spanning the repaired region of the plasmids in the transformants. Ten independent blue transformants containing plasmids formed by fusing vector and insert fragments via 50 bp overlaps were analyzed in this manner, and the results showed that all 20 ends were joined in an error-free manner. Cotransformations using insert fragments containing 25 bp overhangs generated more white colonies than the corresponding experiment employing fragments with 50 bp overhangs. We therefore sequenced the break junctions in ten white colonies to investigate what this background represents. In all cases these transformants contained an insert-free plasmid. These plasmids are most likely formed in a reaction mediated by annealing of the 4 bp overlapping ends produced by MfeI. Next, we analyzed 15 blue transformants from these experiments by diagnostic PCR. These results showed that most transformants contained two types of plasmids, which were either generated by gap repair or by simple religation of the vector fragment (see Supplementary Figure S2). The 15 PCR fragments representing insertion of the *uidA* fragment were all sequenced, and in all cases the fragments were inserted into the vector without introducing any sequence errors in the 30 junctions. All together we conclude that in the absence of NHEJ, in vivo DNA assembly by HR is an accurate and highly efficient process even when it is based on short homology overlapping sequences. However, when the overlapping regions are very short, e.g., 25 bp, competing annealing reactions may start to generate undesired background constructs.

As short overlapping sequences can be easily incorporated into PCR fragments via PCR primer tails, our findings set the stage for *E. coli*-free DNA construction work in filamentous fungi. We therefore next set out to develop a new fungal DNA assembly toolbox, which relies on ordered in vivo HR mediated assembly of PCR fragments. To minimize the risk of alternative annealing reactions, we used 50 bp overhangs as a standard in all of the following experiments unless otherwise specified.

### Flexible Multifragment AMA1 Plasmid Construction by in Vivo DNA Assembly

**Figure 2:**
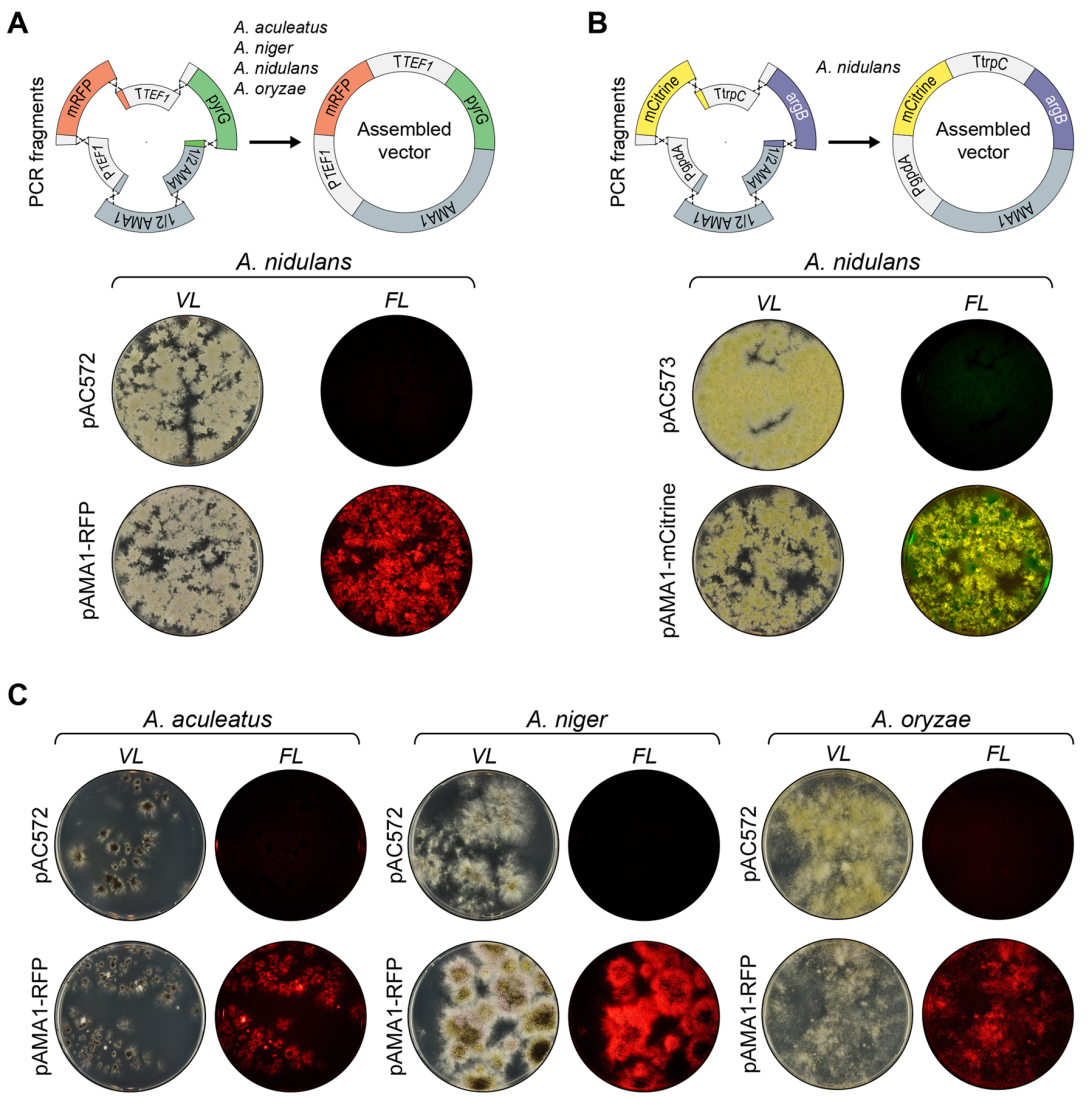
*E. coli* sequence-free (ESF) plasmid construction by in vivo DNA assembly. (A) Top, strategy for the assembly of a fungal pAMA1-*mRFP* plasmid devoid of *E. coli* sequences. Six PCR fragments are joined by in vivo DNA assembly via 50 bp overhangs: *TEF1* promoter, *mRFP* open reading frame, *TEF1* terminator, *pyrG* selectable marker, and two overlapping AMA1 fragments as indicted. Matching fusion sequences are indicated by identical colors. The plasmid parts are not drawn to scale. Below, transformation of the NHEJ deficient *A. nidulans* strain NID2695 with pAC572, an AMA1-*pyrG* control vector, or with the six PCR fragments required for construction of pAMA1-*mRFP* by in vivo DNA assembly as indicated. Transformation plates were imaged at visible light (left) and in a setup detecting red fluorescence (right). (B) Top, strategy for the assembly of the fungal ESF plasmid pAMA1-*mCitrine*. Six PCR fragments are joined by in vivo DNA assembly via 50 bp overhangs: *gpdA* promoter, *mCitrine* open reading frame, *trpC* terminator, *argB* selectable marker, and the two overlapping AMA1 fragments. Below, transformation of the NHEJ deficient *A. nidulans* strain NID2695 with pAC573, an AMA1-*argB* control vector, or with the six PCR fragments required for construction of pAMA1-*mCitrine* by in vivo DNA assembly as indicated. Transformation plates were imaged at visible light (left) and in a setup detecting yellow fluorescence (right). (C) Transformation of NHEJ deficient strains of *A. aculeatus*, *A. niger*, and *A. oryzae* with a AMA1-*pyrG* control vector (pAC572) or with the six PCR fragments required for the construction of the ESF plasmid AMA1-*mRFP* by in vivo DNA assembly. Transformation plates for each species are shown in individual panels as indicated. For each panel, plates representing transformation with pAC572 and with the six PCR fragments required for construction of the plasmid AMA1*-mRFP* are shown in the top and bottom, respectively. In all panels, plates were imaged at visible light (left) and in a setup detecting red fluorescence (right).

In a first application of in vivo DNA assembly, we explored the possibility of using this technology to assemble an episomal fungal vector based on multiple different DNA fragments in a single step. If possible, this will provide a simple setup allowing for swift combinatorial experiments. For example, it would be easy to test the impact of several promoters and terminators on the expression levels from a desirable gene of interest (GOI). Moreover, it would allow constructing smaller fungal vectors that are *E. coli-*sequence-free (ESF). Accordingly, we investigated whether it is possible to assemble a fungal AMA1 based vector containing four different fragments: a selectable fungal marker (*pyrG*), a promoter (P*TEF1*), a GOI encoding the fluorescent reporter protein mRFP, and a terminator (T*TEF1*), by in vivo DNA assembly. Since the AMA1 contains a large inverted repeat^28^ and cannot be synthesized as a single PCR fragment, each repeat was amplified in a separate PCR reaction. Altogether, construction of the new vector therefore requires the assembly of six PCR fragments; see Figure 2A. To ensure ordered assembly, each fusion reaction was performed by a specific sequence tag (see Supplementary Table S3).

To test the efficiency of in vivo plasmid assembly, we cotransformed in triplicate all six PCR fragments into an NHEJ deficient strain of *A. nidulans* (NID2695; see Supplementary Table S2). Encouragingly, transformants were easily obtained (see Figure 2A and Supplementary Figure S3), and more than 90% of the colonies emitted red light when exposed to light with a wavelength that excites mRFP. This result strongly indicates that the PCR fragments containing the *TEF1* promoter, the *mRFP* gene, and the *TEF1* terminator were correctly fused. To test whether the *mRFP* gene-expression cassette (GEC) was incorporated into a *pyrG* based AMA1 plasmid, or into the genome by random integration, six purified transformants were streaked out on nonselective solid media. For all transformants, the fluorescent signal was quickly lost indicating that the *mRFP*-GEC was most likely contained on a *pyrG* harboring AMA1-based plasmid (data not shown). Next, four of the transformants were further examined by Southern blotting (see Materials and Methods) in an experiment designed to investigate whether the *mRFP*-GEC could be released from the putative *pyrG*-AMA1 plasmid. In all four cases, a single band with the expected size could be detected by using a probe specific for the mRFP (see Supplementary Figure S4). Finally, for two transformants, PCR fragments covering all junctions, with the exception of the one in AMA1, were sequenced in order to validate the fusions of the individual DNA fragments. In all cases, fusions were not accompanied by sequence errors (data not shown). Hence, in vivo DNA assembly can be efficiently used to construct a complex AMA1 plasmid in vivo in *A. nidulans*. To determine whether plasmid assembly is restricted to a specific set of DNA fragments, we cotransformed the two AMA1 fragments described above along with four DNA fragments containing a gene encoding mCitrine, a *gpdA* promoter, a *trpC* terminator, and an *argB* marker (Figure 2B) into NID2695. Like above, transformants were readily obtained and the vast majority produced mCitrine; see Figure 2B. Two transformants emitting yellow fluorescence were examined in more detail by diagnostic PCR and sequencing and shown to contain correct and error-free fusions of DNA fragments. Hence, in vivo DNA assembly appears as a versatile tool for multifragment vector construction in NHEJ deficient *A. nidulans*.

In a final set of vector construction experiments, we tested whether efficient in vivo plasmid assembly is a unique feature of *A. nidulans*, or whether other Aspergilli can do it equally well. For this purpose we cotransformed the six fragments necessary for *mRFP pyrG* AMA1 based plasmid assembly (see above) into NHEJ deficient strains of *A. aculeatus* (ACU59), *A. niger* (NIG158), and *A. oryzae* (ORY7) (for strains, see Supplementary Table S2) in triplicate. With all three species, transformants were easily obtained and almost all transformants produced mRFP; see Figure 2C. Like for *A. nidulans*, the mRFP fluorescence signal could be easily lost by transferring purified transformants to solid nonselective medium indicating that the *mRFP*-GEC was harbored on a plasmid rather than being integrated in the genome. In agreement with this, we validated the plasmid by PCR and by sequencing as described above, and no sequence errors were observed (data not shown). Efficient DNA construction mediated by in vivo DNA assembly can therefore also be achieved in these Aspergilli. To our knowledge these are the first examples of fungal AMA1 plasmids devoid of bacterial vector sequences.

### In Vivo DNA Assembly Facilitates Simple Gene-Targeting Substrate Construction and Efficient Gene Deletion

**Figure 3:**
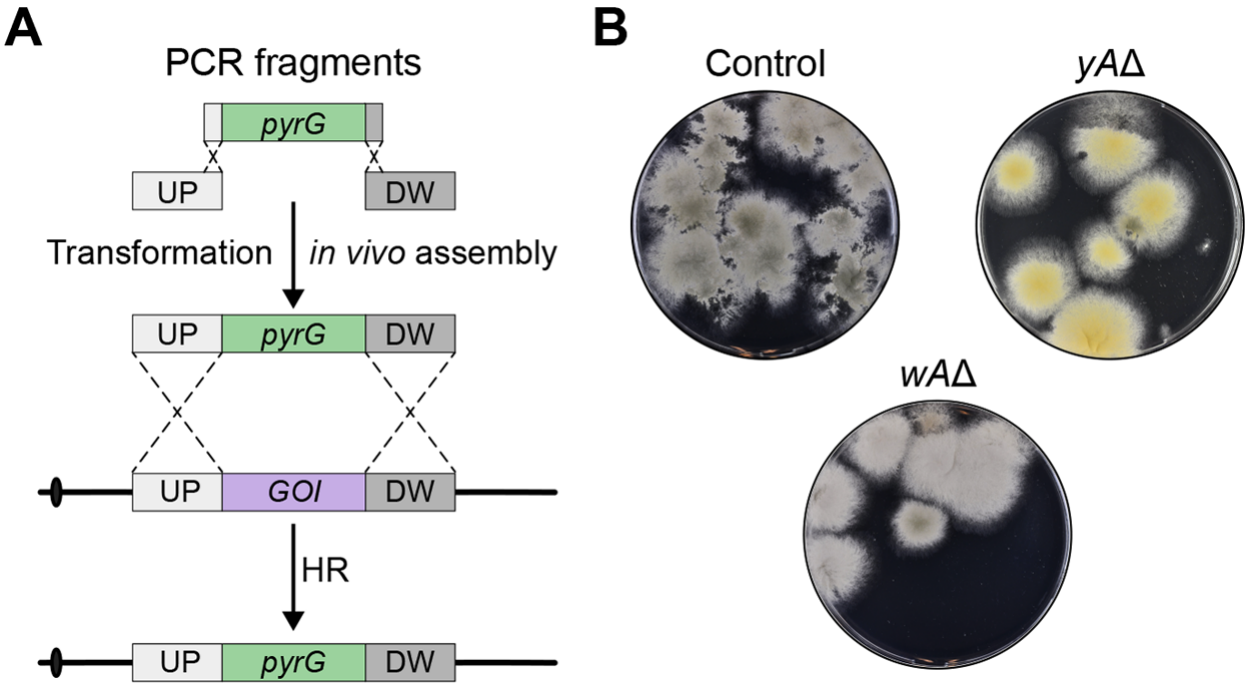
Construction of gene-targeting substrates by in vivo DNA assembly. (A) General strategy to perform a one-step cloning-free gene deletion of a gene of interest, GOI, mediated by in vivo DNA assembly. *pyrG* is used as an example of a selectable marker. Three PCR fragments are combined by in vivo DNA assembly to produce a classical gene-targeting substrate for one-step gene deletion. (B) NID1 was transformed with plasmid pAC572 (plate to the top left) or three PCR fragments: one that contains the *pyrG* marker and two that contain 1000 bp of up- and downstream sequences of *yA* (plate in the top right) or up- and downstream of *wA* (plate to the bottom).

Encouraged by the fact that multiple PCR fragments can easily be assembled in vivo to form episomal vectors, we next envisioned that a gene-targeting substrate for, e.g., gene deletion could be made in the same manner. To test this idea, we investigated whether functional gene-targeting substrates designed for deletion of two color marker genes in *A. nidulans*, *yA* and *wA*,^29,30^ could be assembled in vivo and employed for gene deletion. To this end, deletions of *yA* (a laccase gene) and *wA* (a polyketide synthase gene) produce yellow and white colonies, respectively, which are easy to distinguish from the green wild-type colony color.^29,30^ In each experiment, a PCR fragment containing the selectable *pyrG* marker gene, and two PCR fragments containing 1000 bps of up- and downstream sequences flanking the target gene were generated. The latter were equipped with 50 bp fusion tags matching the *pyrG* marker; see Supplementary Table S3. In vivo DNA assembly of the three fragments generates a classical gene-targeting substrate where the *pyrG* marker is flanked by up- and downstream targeting sequences; see Figure 3A. To assess the efficiency of this gene deletion method, the two sets of PCR fragments were individually cotransformed into an NHEJ deficient strain of *A. nidulans* (NID1) in triplicate. In both experiments, the expected colony color changes were achieved for around 90% of the transformants; see Figure 3B and Supplementary Figure S5. PCR fragments obtained from two yellow and two white colonies were sequenced, demonstrating that they contained the expected deletion of either *yA* or *wA*, respectively. We conclude that gene deletion can easily and efficiently be achieved in *A. nidulans* by an in vivo DNA assembly based method.

### In Vivo DNA Assembly Enables Flexible Gene-Expression Cassette Construction and Subsequent Integration into a Specific Genomic Site

**Figure 4:**
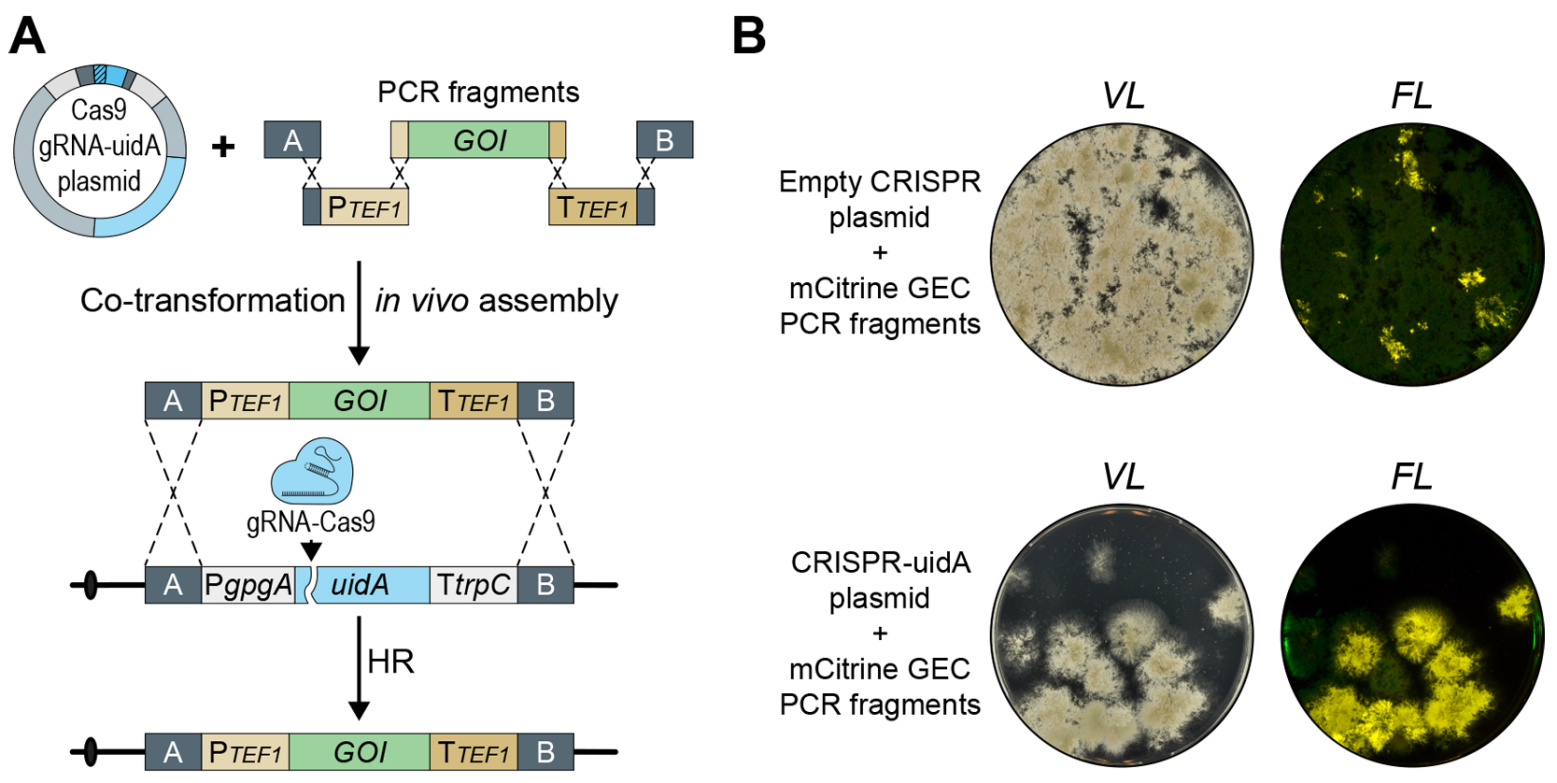
Marker-free chromosomal integration of a gene-expression cassette constructed by in vivo DNA assembly. (A) General strategy to construct a gene-expression cassette, GEC, which can be inserted in a marker-free manner into a specific chromosomal locus via a CRISPR mediated process. Five PCR fragments are merged to form a GEC: two fragments provide the up- and downstream sequences matching the target site (A and B), two fragments provide the promoter and terminator, and the final fragment contains the gene of interest, GOI. In parallel, a CRISPR vector delivers a Cas9/sgRNA nuclease, which introduces a specific DNA DSB at the genomic target site of the GEC. Specific integration of the GEC at the target site is performed by HR. (B) Cotransformation of NID2695 with five PCR fragments, which assemble into an *mCitrine*-GEC for targeting into *uidA* site along with either an empty CRISPR plasmid pFC330 (top) or with a CRISPR vector encoding an sgRNA targeting Cas9 to *uidA* site, pDIV073 (bottom). Colonies on solid medium (left) were imaged by visible light (VL) and in a setup detecting yellow fluorescence (FL).

We have recently published a versatile CRISPR-technology based platform, DIVERSIFY, for gene expression in *A. aculeatus*, *A. nidulans*, *A. niger*, and *A. oryzae*.^31^ In this platform, each starter strain is NHEJ deficient, *pyrG*Δ, and contains a common targeting site COSI-1 for GEC insertion. COSI-1 contains an *uidA* color marker flanked by A and B targeting sequences, and integration of a GEC fused to A and B sequences into COSI-1 can therefore easily be assessed in a blue/white screen as the GEC replaces *uidA*; see Figure 4A. The platform also includes a common *pyrG* based CRISPR-tRNA vector (pDIV073, see Supplementary Table S1), which produces Cas9 and a uidA-sgRNA targeting *uidA* in COSI-1, which facilitates marker-free integration of a GEC into COSI-1.^31^

A bottleneck for using this gene integration platform, e.g., in high-throughput gene-expression experiments, is GEC construction for gene targeting into COSI-1. In a first attempt to use in vivo DNA assembly to simplify selection-free GEC integration into COSI-1, we synthesized five PCR fragments containing the *TEF1* promoter and terminator, the *mCitrine* gene, and A and B targeting sequences. All fragments were equipped with the appropriate 50 bp fusion sequences allowing for orderly assembly into a GEC gene-targeting substrate. Next, the fragments were cotransformed into the *A. nidulans* strain NID2695 either with pDIV073 or with the corresponding empty control CRISPR vector, pFC330 (see Supplementary Table S1).^32^ In three independent trials, we first noted that the numbers of transformants obtained with pDIV073 were much lower than with the control vector pFC330. The lower number of transformants is probably caused by cell death due to unrepaired Cas9 induced DNA DSBs.^25,33^ If so, these results indicate that Cas9/*uidA*-sgRNA efficiently cuts *uidA*, but that formation of the integrative GEC cassette, which is necessary for repair, is limiting. In agreement with this view, we observed that the number of transformants increased with increasing concentrations of GEC parts (compare Figure 4B and Supplementary Figure S6A). Importantly, approximately 65% of the transformants obtained with pDIV073 appear to be cases where the integrative *mCitrine*-GEC was assembled and used for repair of a DNA DSB in *uidA*. Hence, they emitted yellow fluorescent light and did not display β-glucuronidase activity (Figure 4B and Supplementary Figure S6B). For three of these transformants, PCR fragments covering the expression site and the GEC were sequenced to demonstrate that this phenotype was due to replacement of *uidA* with *mCitrine*. This analysis did not identify any mutations, showing that the fidelity of integrative GEC assemble is high. A small number of the transformants appeared to have brighter fluorescence signal; however, we note that these colonies also appeared blue on the X-Gluc plates, indicating that the *uidA* gene was not replaced and that they may therefore represent aberrant integration events. Lastly, we note that even with the empty vector, yellow fluorescent colonies were obtained and these were also devoid of β-glucuronidase activity. These transformants were rare, less than 5% of the total number of transformants, and likely represent rare regular gene-targeting events obtained without selection. In parallel, we performed a similar integration experiment in triplicate using the same set of DNA fragments, except that the gene encoding mCitrine was substituted for one encoding mRFP. Cotransformation of this set of DNA fragments into NID2695 produced fluorescent transformants devoid of β-glucuronidase activity with the same efficiency as those obtained with the mCitrine set (see Supplementary Figure S7), hence showing that other genes can also be integrated into COSI-1 in this manner.

The fact that some colonies showed aberrant phenotypes prompted us to randomly pick four fluorescent colonies from each experiment, purify them, and analyze them by Southern blotting. With the *mRFP*-GEC, this analysis showed that all four transformants produced the predicted pattern expected from a single integration into COSI-1, Supplementary Figure S8A,B. With the *mCitrine*-GEC, all four transformants contained an mCitrine gene in COSI-1; however, two of the transformants appeared to contain an additional *mCitrine* gene copy; see Supplementary Figure S8C,D. To investigate this phenomenon in more detail, we sequenced the genome of the latter two transformants using Nanopore technology. These analyses demonstrated that the *mCitrine*-GEC has recombined with the CRISPR plasmid in reactions mediated by the *TEF1* promoter and terminator sequences that were used to control expression of *cas9* as well as of *mCitrine*.

### In Vivo DNA Assembly Simplifies CRISPR Mediated Genetic Engineering

**Figure 5:**
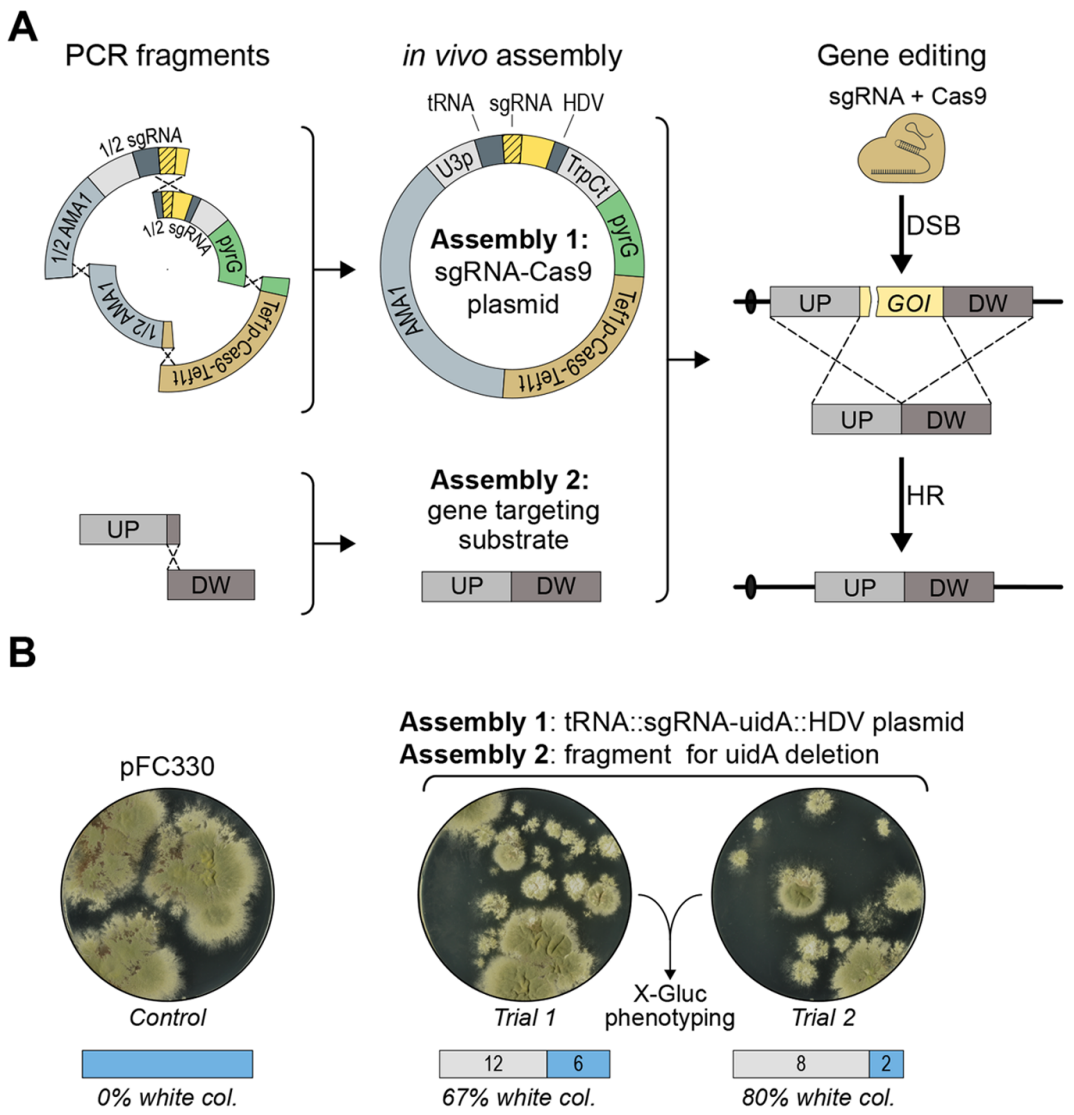
Cloning- and marker-free gene deletion. (A) An ESF-CRISPR vector containing a unique sgRNA expression cassette and a marker-free gene-targeting substrate for gene deletion are constructed in parallel in two independent in vivo DNA assembly reactions. The ESF-CRISPR vector is assembled from four different PCR fragments. Importantly, two of the PCR fragments are fused via tags that include the variable moiety of the sgRNA sequence to produce the unique sgRNA expression cassette. Since the fusion tags are included in the primer tails, the coding sequence of the sgRNA gene can be easily reprogrammed to match new target sites. The marker-free gene deletion substrate is formed by fusing two PCR fragments containing up- and downstream sequences of the target gene. After in vivo DNA assembly of the two constructs, Cas9 introduces a specific DNA DSB in the target gene, and repair of this break using the gene-targeting substrate produces the desired deletion. (B) Transformation of NID1 with pFC330 (left), and with the two PCR fragments required for the assembly of the gene-targeting substrate for deletion of *uidA* and the four fragments required to build the *uidA* ESF-CRISPR vector (right).

CRISPR technologies are increasingly used in fungal genetic engineering.^34−37^ Many CRISPR methods are based on in vivo assembly of the CRISPR nuclease and the sgRNA, and typically require *E. coli*-based construction of one or more vectors that can deliver the specific sgRNA and most often also the CRISPR nuclease.^38^ To bypass the *E. coli* cloning step, we envisioned the possibility of using in vivo DNA assembly for the construction of a vector that delivers Cas9 and an sgRNA. However, our current designs for vector based sgRNA production involve sgRNA expression cassettes containing repeated sequences,^25,32^ and homology mediated fusion reactions introducing new sgRNA sequences into these cassettes would therefore be prone to alternative assembly reactions involving these repeats. To avoid this problem, we designed a new sgRNA setup, *tRNA::sgRNA::HDV*, which does not contain repeated DNA sequences. Using an sgRNA we have previously used to mutate *yA*, yA-sgRNA1,^25^ we constructed an ESF-CRISPR vector pAC1935 (see Supplementary Figure S9A and Supplementary Table S1) containing the founding version of this type of sgRNA expression cassette flanked by the *Af_U3* promoter and *trpC* terminator. In a set of control experiments we demonstrated that pAC1935 can be efficiently used for gene editing and that is possible to reprogram the sgRNA expression cassette of pAC1935 by merging four PCR fragments by DNA fusion (see Supplementary Figure S9B and Figure 5A). Due to the size of the programmable sgRNA section of the vector, we used 60 bp overhangs to generate and insert the sgRNA coding sequence into the plasmid.

In a final experiment we tested whether CRISPR mediated marker-free gene-targeting, which is solely based on PCR fragments, can be performed via two in vivo DNA assembly reactions performed in parallel; see Figure 5A. In one reaction, a marker-free gene-targeting substrate for deletion of the *uidA* reporter gene in COSI-1 was made by fusing two PCR fragments. In the other reaction, an ESF-CRISPR vector expressing an sgRNA targeting *uidA* was constructed via the in vivo assembly of four PCR fragments. Three independent transformations were performed, each of which resulted in 15–20 transformants; see Figure 5B and Supplementary Figure S10. All transformants were selected for blue/white color assessment, and the results indicate that the combined efficiency of in vivo gene-targeting substrate assembly and CRISPR mediated gene deletion ranges from 45–80%. To validate that *uidA* activity was indeed lost due to the desired gene deletion event, we selected one white transformant for each transformation for diagnostic PCR analysis. All three transformants produced the expected PCR fragments, which were subsequently sequenced. This analysis demonstrated that no errors at sequence levels were incorporated as the result of CRISPR mediated gene deletion (data not shown).

## Discussion

We have investigated the possibility of fusing DNA fragments via overlapping sequences in Aspergilli by HR. In a first set of experiments we investigated the influence of NHEJ on HR mediated DNA-end fusing in a plasmid gap repair assay. In wild-type *A. nidulans* strains, we observed that with small sequence overlaps (25–200 bps) most plasmids were the result of NHEJ events, whereas with large sequence overlaps (400–1600 bps) they were formed by HR. Hence, it appears that NHEJ and HR compete for DNA ends and that the outcome of the competition depends on the length of the overlapping sequences. Importantly, in the absence of NHEJ, DNA fragments are joined almost solely by HR even when the sequence overlaps are very short. Moreover, the efficiency of gap repair is almost independent of the length of the overlapping sequences. These results suggest that DNA ends that would be joined by NHEJ in a wild-type strain are not lost from the population, but rather they are channeled into HR repair. These results contrast observations showing that efficient gene targeting even in NHEJ deficient filamentous fungi depends on long ≥500 bps targeting sequences,^19−22^ and suggest that fusion of DNA fragment ends appears to be mechanistically different from gene targeting. To this end we note that gene targeting requires strand invasion into intact DNA in chromatin.^39^ In contrast, end fusion in our gap repair assay most likely involves nonchromatin DNA. Moreover, if both DNA fragment ends are processed by HR nucleases to produce 3′-ssDNA tails during gap repair, end fusion may occur by single-strand annealing,^39^ which is mechanistically much simpler as compared to HR involving strand invasion.

We have shown that 25 bp sequence overlaps are sufficient to fuse ends in two different DNA fragments correctly and that 4 bp overlaps are enough to mediate efficient fusion of ends if they are present in the same DNA fragment. These results demonstrate that even very short sequences can be used to join DNA fragments in a guided manner. The results also emphasize that shortening of the overhangs used for fragment assembly may increase the levels of undesirable background due to competing assembly reactions. In the *uidA* gap repair experiment, the tipping point seems to be reached when the sequence overlaps of the insert fragment are reduced to 25 bp. Importantly, we speculate that if the gap repair experiment had been performed with a vector fragment containing no complementary sequences in its ends, then 25 bp, and perhaps even shorter, sequence overlaps would be sufficient to produce transformants that solely result in the correct plasmid. On the other hand, given the high efficiency of DNA assembly, it is generally advisable to examine the individual DNA components in the planned DNA assembly experiment for repeated sequences in the fragment ends, especially if short overlaps are used to fuse the DNA fragments.

Efficient HR mediated end-fusions are not restricted to *A. nidulans* as they can also be performed in *A. aculeatus*, *A. oryzae*, and *A. niger* (see Figure 2C). Moreover, we have shown that several fragments—we tested up to six—can be correctly and orderly assembled into larger structures with high efficiency. Interestingly, CRISPR mediated gene targeting in *Penicillium chrysogenum* has been performed with gene-targeting substrates containing short targeting sequences (60–100 bps).^24^ Moreover, it was recently shown that DNA fragment ends with 100 bp^40^ overhangs were joined efficiently in *P. rubens*. We therefore speculate that DNA assembly via very short 20–50 bp overhangs may be efficient in a wide range of filamentous fungi.

The high efficiency of DNA end fusion prompted us to develop a novel fungal in vivo DNA assembly toolbox, which can be used to facilitate a wide range of typical strain engineering processes; see the Graphical Abstract. In one set of experiments, we have demonstrated that our technology set the stage for plasmid construction directly in filamentous fungi, hence eliminating the need for an *E. coli* cloning step as well as the need for sequences that allow for selection and replication in *E. coli*. We note that this technique eliminates a potential need to account for products that could result from bacterial marker genes, e.g., beta lactamase. Moreover, we speculate that the smaller size of the resulting ESF vectors may increase their stability. In addition to ESF vectors, we also demonstrated that gene-targeting substrates for gene deletions and gene insertions can be efficiently assembled from DNA fragments generated from a single round of PCR. Lastly, we introduce a novel CRISPR tool based on in vivo DNA assembly that sets the stage for marker- and cloning-free genetic engineering as we demonstrate that a DNA fragment encoding an sgRNA can be functionally fused into a CRISPR vector. Moreover, in this experiment we also demonstrate that it is possible to assemble several constructs in parallel in vivo. In the present cases, we assembled a GEC and ESF-CRISPR vector simultaneously in vivo. As a result the GEC was successfully inserted into an expression platform located in the genome via a CRISPR mediated step. With this subset of experiments we demonstrate that in vivo DNA assembly can be used to efficiently perform a wide range of strain construction tasks, and we feel confident that the repertoire will be expanded in the future. In the different experiments, we have fused more than 40 fragment ends together for different purposes and sequenced at least two independent reactions for each case without observing any errors in the fusion regions, and in vivo DNA assembly therefore does not appear to introduce significant levels of sequence errors at the fusion points. We note that in the case of *mRFP* and *mCitrine* gene insertions, an additional copy of the *mCitrine* gene was present in the genome of two of the eight clones analyzed, indicating that aberrant fusion or integration events may take place. Hence, like for constructs made by regular *E. coli* cloning, thorough validation of constructs made by in vivo DNA assembly is therefore recommended.

We stress that all construction work presented here is mediated by the assembly of PCR or synthetic fragments. Since sequences in PCR tails determine how the fragments are combined, it is easy to recruit new building blocks from larger collections, e.g., the recently published synthetic biology toolkit for filamentous fungi.^41^ Moreover, a barcode labeling for individual parts can also be implemented as part of a primer. Importantly, the efficiency and flexibility of the in vivo DNA assembly methods make them highly suitable for large scale experiments that depend on high-throughput strain construction.

## Materials and Methods

### Strains and Media

All plasmids were propagated in *Escherichia coli* strain DH5α. Solid (2% agar) or liquid Luria broth (LB) medium supplemented with 100 μg/mL ampicillin was used as growth medium.

Aspergilli strains used in this study are listed in Supplementary Table S2. All strains were cultivated using liquid or solid (2% agar) minimal medium (MM) (1% glucose, 1× nitrate salt solution,^42^ 0.001% Thiamine, 1× trace metal solution),^43^ which was supplemented with 10 mM uridine (uri), 10 mM uracil (ura), and/or 4 mM l-arginine (arg) when required. To perform blue/white screening, solid MM was supplemented with 0.115 mM X-Gluc (Thermo Fisher Scientific). Transformation medium (TM) was prepared as MM, except for glucose, which was substituted with 1 M sucrose.

### PCR Fragment Amplification and Plasmid Construction

All PCR reactions, restriction-enzyme digestions, ligations, and DNA purifications by kits were carried out according to the manufacturer’s instructions unless otherwise specified. The *An*_P*gpdA-uidA-An*_T*trpC* fragment encoding the full *uidA* reporter cassette as well as truncated *uidA*-containing PCR fragments for the gap repair assay were amplified from pDIV083 using primers (Integrated DNA Technologies, IDT) in the pairs listed in Supplementary Table S3. For these reactions, PhilisaFAST (Streck) was used with 0.4 μM primers and the following reaction settings on BioRad PCR cyclers: 95 °C for 3 min; followed by 35 cycles of 30 s at 95 °C, 30 s at 64 °C with touchdown of −0.2 °C per cycle decrease, and 72 °C for 150 s; and 10 min at 72 °C. Fragments were purified via the Zymoclean Gel DNA recovery kit (Zymo Research).

For amplification of the remaining fragments for transformations, validation of strains in AMA1 assembly experiments, gene deletion and gene integration experiments, as well as amplifying the probes for Southern blots, PCR reactions were performed using proofreading Phusion U polymerase (Thermo Fisher Scientific) and primers at 0.5 μM listed in Supplementary Table S3. PCR fragments were purified using NucleoSpin Gel and PCR Clean-up kit (Macherey-Nagel).

USER cloning^44,45^ was used to assemble the plasmids listed in Supplementary Table S1. All plasmids were purified using GenElute Plasmid Miniprep Kit (Merck). Specifically, the *An*_P*gpdA-uidA-An*_T*trpC* fragment was inserted in the USER-compatible pAC573 harboring *AMA1* and *argB*, confirmed by BspEI (New England Biolabs, NEB) digestion and gel electrophoresis. The resulting vector, pAC1688, was opened by High-Fidelity MfeI (NEB) treatment at two restriction sites resulting in a loss of a fragment containing parts of P*gpdA* and *uidA* gene; see main text and Supplementary Figure S1. Gel purified vector backbone was treated with T4 DNA ligase (NEB) to generate a vector pAC1767 with a single MfeI cut site, which was propagated for amplification and verified by PCR (Supplementary Table S3). For the gap repair assay, pAC1767 was linearized with High-Fidelity MfeI and purified by column precipitation using illustra GFX PCR DNA and Gel Band Purification Kit (GE Healthcare).

For in vivo DNA assembly CRISPR experiments the plasmid pAC1935 was constructed by fusing three USER compatible PCR fragments AMA1 part2-*Af*_*U3*p::*tRNA*::*yA*-sgRNA1, *sgRNA*::*HDV*::*An*_T*trpC*-*cas9*-*Af*_*pyrG*-*ori*, and *ori*-*ampR*-AMA1 part 1. The plasmid was confirmed by *Eco*RI/PacI (NEB) digestion and sequencing.

ESF-CRISPR PCR fragments for in vivo assembly were amplified using pAC1935, a total of four fragments were required for each experiment AMA1-part1, AMA1-part2::*sgRNA*-part1, sgRNA-part2::*Af*_*pyrG*, and *cas9*. The gene-targeting substrate was merged by in vivo DNA assembly of two PCR fragments encoding up- and downstream sequences flanking *uidA*. Primers for all CRISPR experiments can be found in Supplementary Table S3.

### Fungal Transformations

Protoplasts were generated according to the protocol described by Nielsen et al.^15^ For each transformation, protoplasts were mixed with the plasmid and/or purified PCR fragments, and added to 150 μL of PCT solution (50% w/v PEG8000, 50 mM CaCl_2_, 20 mM Tris, 0.6 M KCl, pH 7.5). The mix was incubated for 10 min at room temperature, followed by addition of 250 μL of transformation buffer (1.2 M sorbitol, 50 mM CaCl_2_·2 H_2_O, 20 mM Tris, 0.6 M KCl, pH 7.2), and plating on TM plates with appropriate supplements. All transformation plates were incubated at 30 °C, except for *A. nidulans* transformations, which were incubated at 37 °C.

For transformations in the gap repair experiment, 0.02 pmol of linearized pAC1767 was combined with linear repair fragments in a stoichiometric ratio of 1:10 including a positive control with circular pAC1688 encoding a functional *uidA*, and a negative control with linearized pAC1767 without repair fragments. In AMA1 assembly experiment 0.2 pmol of each PCR fragment was used for transformation, and as a control 0.5 μg of either pAC572 or pAC573 plasmid was used. For gene deletion transformations, strains were transformed with 0.8 pmol of each PCR fragment or 0.5 μg of pAC572 plasmid as a control. For transformations in gene integration experiment 0.5 μg of pDIV073 or pFC330 (control vector) was cotransformed with either 0.2 pmol (one trial) or 0.8 pmol (two trials) of each PCR fragment. Lastly, in the marker-free gene deletion and ESF-CRISPR experiments strains were cotransformed with 0.8 pmol of each PCR fragment.

### Strain Validation

To validate the strains in AMA1 assembly, gene deletion, and gene integration experiments, gDNA was extracted by harvesting the biomass from solid media, and mixing with 500 μL of lysis buffer (2% Triton X-100, 1% SDS, 100 mM NaCl, 10 mM TrisHCl, 1 mM EDTA, 200 mM LiAc) and 200 μL of 0.5 mm glass beads. The samples were homogenized in Thermo Savant Bio 101 FastPrep FP120 cell disruptor at speed 4 for 40 s, followed by centrifugation at 12 000*g* for 5 min. 150 μL of supernatant was transferred to a new tube and mixed with 15 μL of 5 M NaCl and 400 μL of ice-cold 96% ethanol. After centrifugation at 10 000*g* for 3 min, supernatant was aspirated and the samples were dried at 50 °C for 30 min, and subsequently dissolved in 200 μL MiliQ water. Extracted gDNA was used as a template for diagnostic PCR, followed by purification of the fragments as described above. Purified PCR fragments were sent for sequencing (Eurofins Genomics) with primers listed in Supplementary Table S3. For analysis of the blue colonies derived from the gap repair assay, the tissue-PCR approach was as described in Nødvig et al.^32^

### Fluorescence Photography

To confirm the production of mCitrine and mRFP, solid media plates were examined for yellow and red fluorescence with the setup described by Vanegas et al.^26^ The exposure time for both YFP and RFP filters was 0.25 s.

### Southern Blot

Genomic DNA was isolated using FastDNA SPIN Kit for Soil (MP Biomedicals, USA) according to the manufacturer’s instructions. For each strain with marker-free gene integration, 2 μg of gDNA was digested with EcoRV enzyme (NEB) and for each plasmid assembly strain, 1.5 μg of gDNA was digested with *Bgl*II and NotI enzymes (NEB). Blotting was performed as described by Sambrook and Russell.^46^ Probes were generated using primers listed in Supplementary Table S3, and plasmids pDIV088 and pDIV089 as PCR templates (see Supplementary Table S1). The PCR for the mCitrine probe yielded a fragment of 540 bp, and for mRFP, 600 bp. Probes were labeled with the Biotin DecaLabel DNA Labeling Kit (Thermo Scientific), and the Biotin Chromogenic detection kit (ThermoFisher Scientific) was used for detection.

### Nanopore Sequencing

For genomic DNA extraction, *A. nidulans* conidia were inoculated into 250 mL shake flasks containing 50 mL of YPD media supplemented with 10 mM ura, 10 mM uri, and 4 mM arg. The flasks were incubated for 48 h at 37 °C and 150 rpm. Mycelia were ground in a mortar with liquid nitrogen followed by resuspension in lysis buffer composed of 350 mM sorbitol, 30 mM Tris-HCl (pH 9), 55 mM EDTA (pH 8), 1 M NaCl, 27 mM CTAB, 0.45% (m/v) sarkosyl, 0.1% (m/v) PVP, and 100 μL Proteinase K. Samples were washed with phenol:chloroform:isoamyl alcohol (25:24:1) twice, followed by chloroform wash (twice) and ethanol precipitation. gDNA was treated with RNase and centrifuged, and then washed with 70% ethanol and allowed to air-dry. gDNA was resuspended in DNase-free MiliQ water, and the quality control was performed using 1% agarose gel, Nanodrop, and Qubit. Nanopore data were generated using the SQK-RBK004 kit and a 9.4.1 flowcell on a MinION machine. Basecalling and demultiplexing was done with the high accuracy Guppy v.5.0.17 + 99baa5b model on a GPU enabled computer.

## Supporting Information Available

Supplementary figures demonstrating (1) construction of plasmid pAC1767, (2) diagnostic PCR results for gap repair experiment with 25 bp overhangs, (3) plasmid assembly experiments, (4) Southern blot analysis of pAMA1-*mRFP*, (5) gene deletion experiments, (6) assembly and integration of *mCitrine*-GEC, (7) assembly and integration of *mRFP*-GEC, (8) Southern blot analysis of GEC insertion strains, (9) validation of tRNA::sgRNA::HDV setup, (10) marker-free gene deletion experiments; Supplementary tables listing (1) plasmids, (2) strains, (3) primers (PDF)

## Abbreviations

COSI-1 site: common synthetic gene integration site
CRISPR: clustered regularly interspaced short palindromic repeat
DSB: double-strand break
ESF: *E. coli*-sequence-free
GEC: gene-expression cassette
GOI: gene of interest
HR: homologous recombination
NHEJ: nonhomologous end-joining.
