# Supplementary material for: A Versatile in Vivo DNA Assembly Toolbox for Fungal Strain Engineering
Source: ACS Synth Biol. 2022 Sep 20;11(10):3251–63. doi: 10.1021/acssynbio.2c00159 (PMC9594312; doi:10.1021/acssynbio.2c00159)
Supplement: Supplementary file 1 — sb2c00159_si_001.pdf [file sb2c00159_si_001.pdf]

Supplementary Information

A versatile in vivo DNA assembly toolbox for fungal strain engineering

Zofia Dorota Jarczynska[1,†], Katherina Garcia Vanegas[1,†], Marcus Deichmann[1], Christina Nørskov Jensen[1], Marouschka Jasmijn Scheeper[1], Malgorzata Ewa Futyma[1], Tomas Strucko[1], Fabiano Jares Contesini[1], Tue Sparholt Jørgensen[2], Jakob Blæsbjerg Hoof[1], Uffe Hasbro Mortensen[1,*]

[1]Eukaryotic Molecular Cell Biology, Section for Synthetic Biology, Department of Biotechnology and Biomedicine, Technical University of Denmark, Kongens Lyngby, Denmark

[2]The Novo Nordisk Foundation Center for Biosustainability, Technical University of Denmark, Kongens Lyngby, Denmark

[†]Joint authors

[*]Corresponding author, um@bio.dtu.dk

The Supplementary Information contains 10 figures and 3 tables.

Figures:

Supplementary Figure S1 Construction of plasmid pAC1767.

Supplementary Figure S2 Gap repair of plasmid pAC1767 in the presence of a DNA repair fragment with 25 bp overlapping sequences to the plasmid ends.

Supplementary Figure S3 Plasmid construction by *in vivo* assembly.

Supplementary Figure S4 Southern blot analysis of *A. nidulans* strains harboring pAMA1-*mRFP*.

Supplementary Figure S5 *E. coli* cloning-free gene deletion mediated by *in vivo* assembly.

Supplementary Figure S6 Marker-free chromosomal integration of a *mCitrine*-GEC cassette by *in vivo* assembly.

Supplementary Figure S7 Marker-free chromosomal integration of a *mRFP*-GEC cassette by *in vivo* assembly.

Supplementary Figure S8 Southern blots of GEC insertion strains generated by *in vivo* assembly.

Supplementary Figure S9 Validation of the tRNA::sgRNA::HDV setup for sgRNA release.

Supplementary Figure S10 Cloning- and marker-free gene deletion by *in vivo* assembly.

Tables:

Supplementary Table S1 Vectors used in this study.

Supplementary Table S2 Strains used in this study.

Supplementary Table S3 List of primers used in this study.

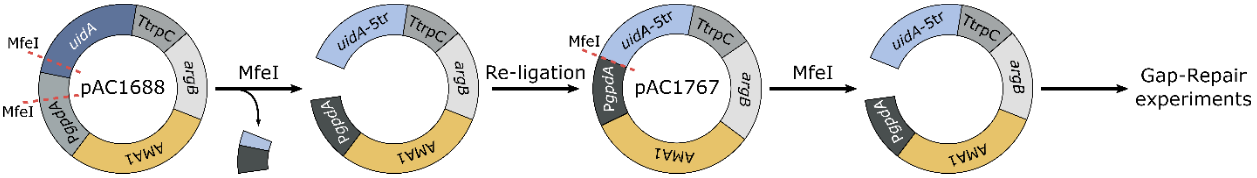

Supplementary Figure S1 Construction of plasmid pAC1767. To prepare the vector for gap-repair experiments, pAC1688 was treated with MfeI. This resulted in excision of a fragment containing the 3'-part of the promoter and the start of the *uidA* gene. The resulting vector backbone was purified from agarose gel and re-ligated by T4 DNA ligase to give pAC1767 with one functional MfeI site and a non-functional *uidA* gene. After propagation of pAC1767, it was digested with MfeI to produce a completely linearized vector applicable for gap repair experiments.

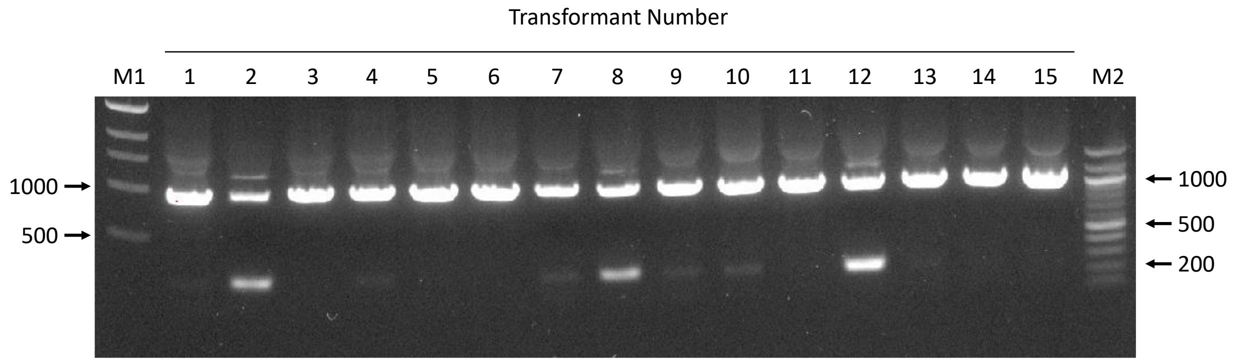

Supplementary Figure S2 Gap repair of plasmid pAC1767 in the presence of a DNA repair fragment with 25 bp overlapping sequences to the plasmid ends. 15 blue transformants were analyzed for the presence of the insert by diagnostic PCR using genomic DNA from the individual transformants as template. Repair mediated by the DNA repair fragment produces a band of 1 kb and simple plasmid religation produces a band of 200 bp. M1 and M2 represent 1 kb and 100 bp markers, respectively.

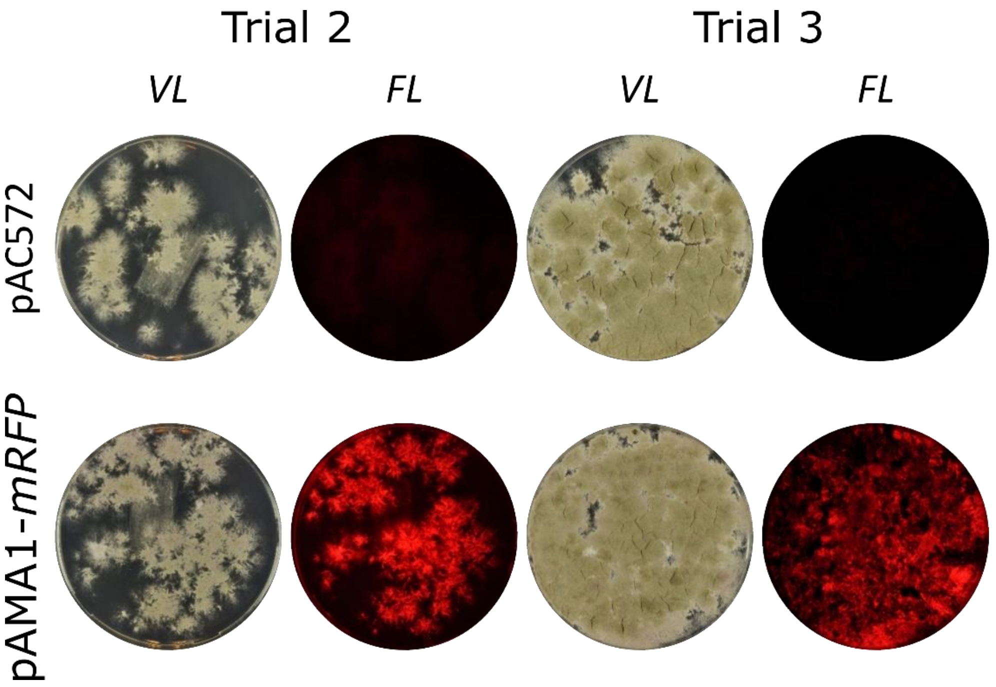

Supplementary Figure S3 Plasmid construction by *in vivo* assembly. Transformation of the NHEJ deficient *A. nidulans* strain NID2695 with pAC572, an AMA1-*pyrG* control vector, or with the six PCR fragments required for the *in vivo* assembly of pAMA1-*mRFP*, trial 2 and 3. For details, see main text. Transformation plates were imaged at visible light (VL) and in a setup detecting red fluorescence (FL).

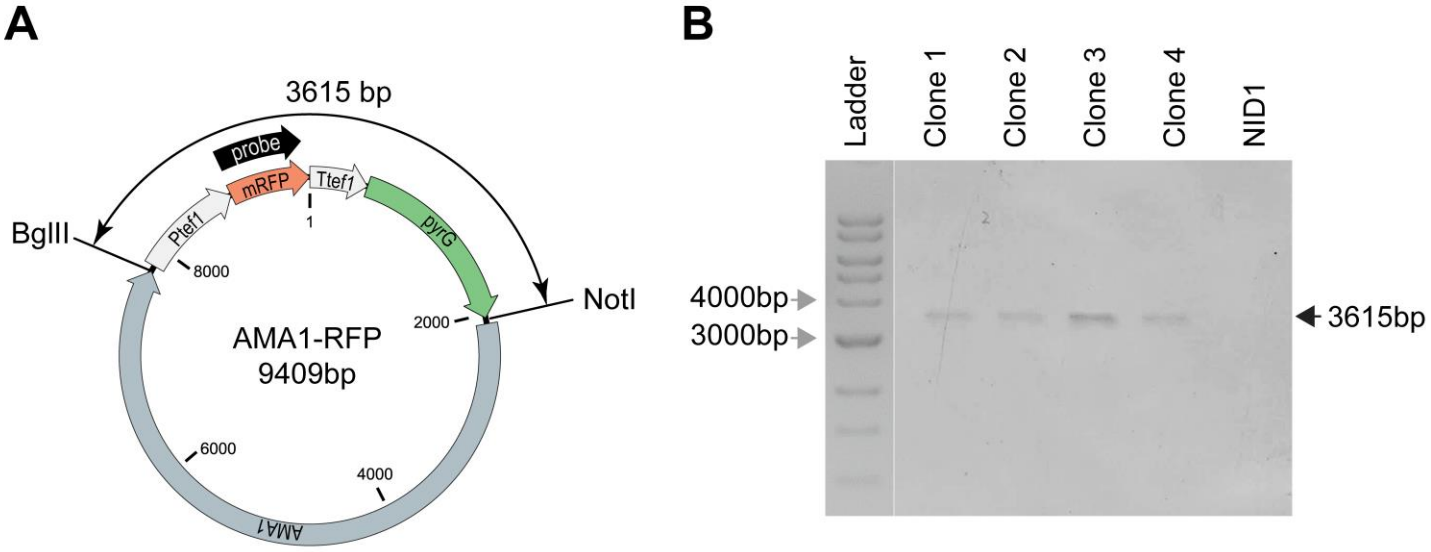

Supplementary Figure S4 Southern blot analysis of *A. nidulans* strains harboring pAMA1-*mRFP*. **(A)** Extracted genomic DNA was digested with BglII and NotI restriction enzymes liberating a band of 3615 bp if the vector was correctly assembled. **(B)** Samples were loaded in the following order: 1 kb ladder, four different purified transformants (clones 1-4) randomly selected from the transformation plate, and the control strain NID1 (no plasmid). All the transformants showed the correct band size for proper plasmid assembly.

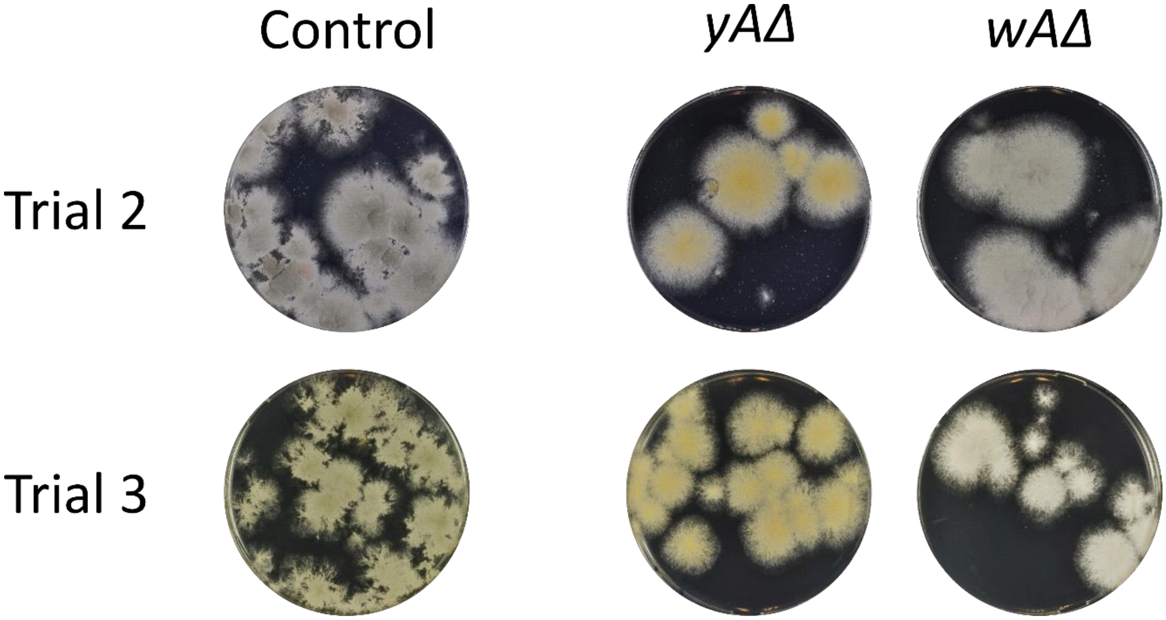

Supplementary Figure S5 *E. coli* cloning-free gene deletion mediated by *in vivo* assembly. NID1 was transformed with plasmid pAC572 (control) or three PCR fragments: one that contains the *pyrG* marker and two that contain 1000 bp of up- and downstream sequences of *yA* (*yAΔ*) or up- and downstream sequences of *wA* (*wAΔ*). Shown are the results of trial 2 and 3 (for trial 1, see main text).

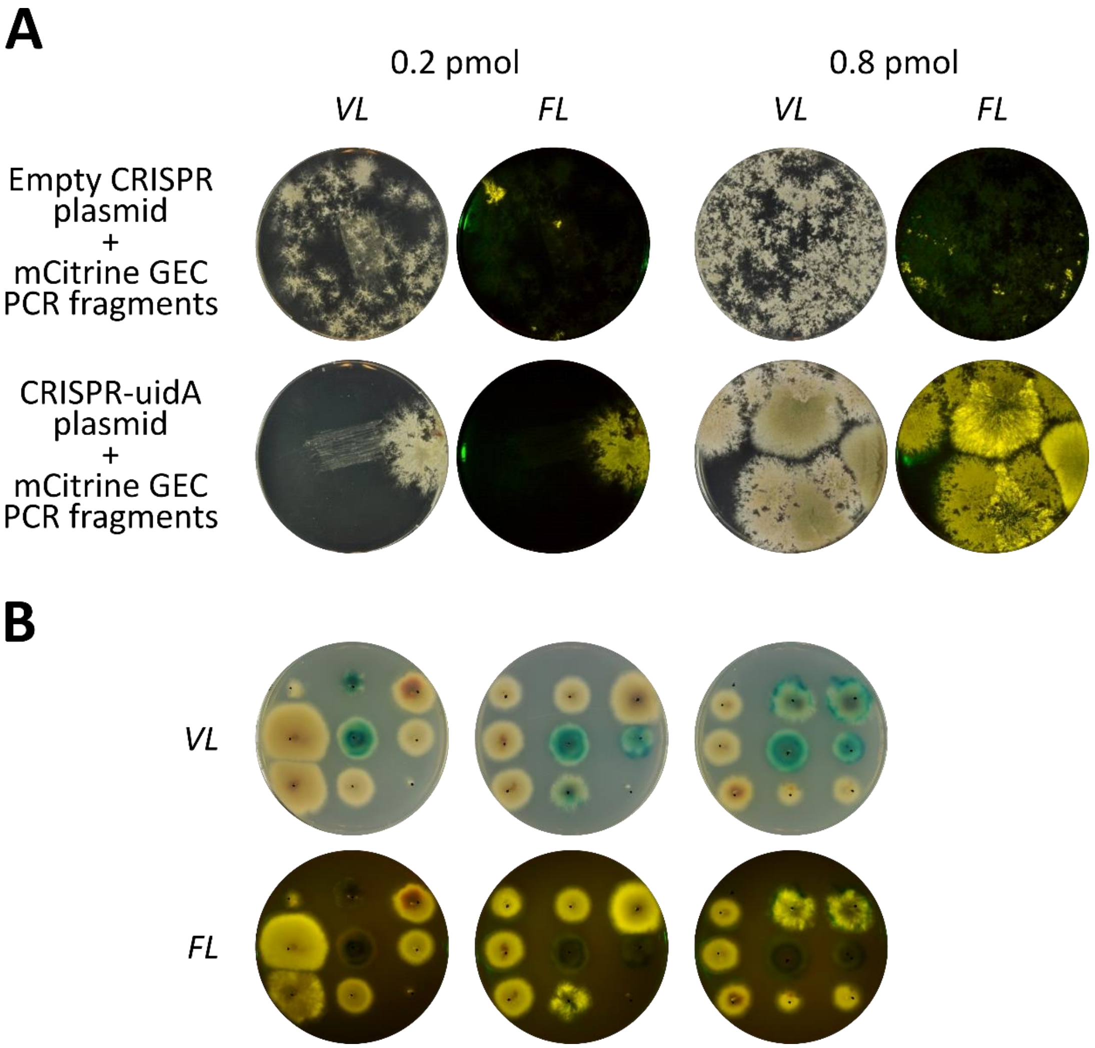

Supplementary Figure S6 Marker-free chromosomal integration of a *mCitrine*-GEC cassette by *in vivo* assembly. NID2695 contains an artificial insertion site for gene expression, COSI-1 (1). COSI-1 contains the color marker *uidA*. In our system, a GEC is inserted into COSI-1 at the expense of *uidA*. Hence, correct targeting of COSI-1 in NID2695 changes its color from blue to white on X-Gluc plates. **(A)** Transformation of NID2695 with five PCR fragments, which assemble *in vivo* into an *mCitrine*-GEC for targeting into the artificial COSI1-1 site, along with an empty CRISPR plasmid pFC330 (top) or with a CRISPR vector encoding an sgRNA targeting Cas9 to *uidA* site, pDIV073 (bottom). In one trial (left) 0.2 pmol of each PCR fragment was used for transformation, and in second trial (right) the amount was increased to 0.8 pmol. Colonies on solid medium were imaged by visible light (VL) and in a setup detecting yellow fluorescence (FL). **(B)** *A. nidulans* strains expressing *mCitrine*-GEC on X-Gluc media. The randomly selected colonies were transferred to solid MM media containing X-Gluc. In the middle of each plate, a control NID2695 strain expressing functional *uidA* gene, but not *mCitrine*, was positioned for comparison. Plates were imaged at white light (VL) and in a setup detecting yellow fluorescence (FL). White colonies which

also has yellow fluorescence signal represent the successful replacement of *uidA* by *mCitrine*-GEC. Blue colonies with no fluorescence signal represent the colonies in which *mCitrine*-GEC was not integrated into the *uidA* locus. Blue colonies with fluorescence signal represent heterokaryons.

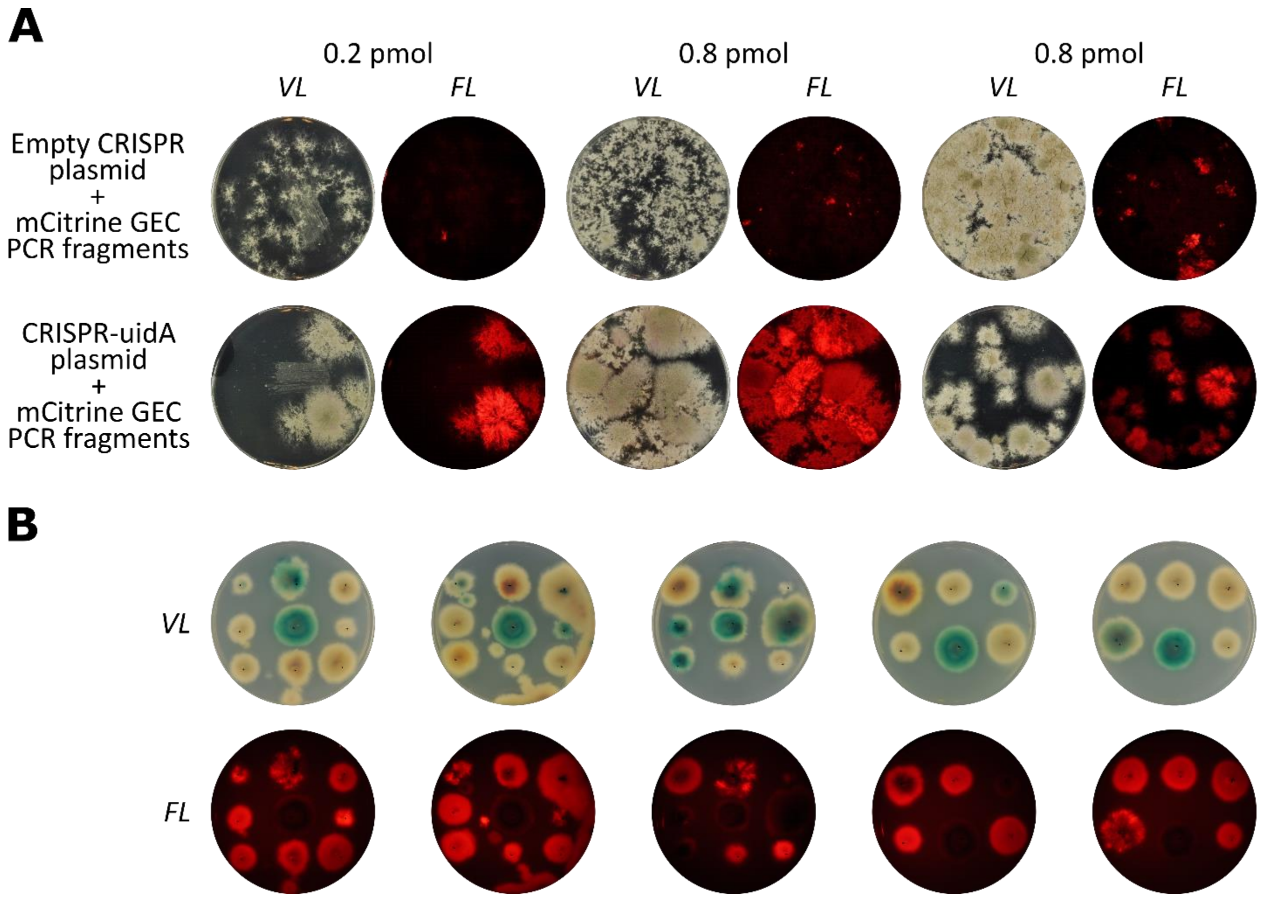

Supplementary Figure S7 Marker-free chromosomal integration of a *mRFP*-GEC cassette by *in vivo* assembly. **(A)** Transformation of NID2695 with five PCR fragments, which assemble *in vivo* into an *mRFP*-GEC for targeting into the artificial COSI1-1 (1) site containing *uidA*, along with an empty CRISPR plasmid pFC330 (top) or with a CRISPR vector encoding an sgRNA targeting Cas9 to *uidA* site, pDIV073 (bottom). In one trial (left) 0.2 pmol of each PCR fragment was used for transformation, and in second and third trials (middle and right) the amount was increased to 0.8 pmol. Colonies on solid medium were imaged by visible light (VL) and in a setup detecting red fluorescence (FL). **(B)** *A. nidulans* strains expressing *mRFP*-GEC on X-Gluc media. The randomly selected colonies were transferred to solid MM media containing X-Gluc. In the middle of each plate (or middle of the bottom row for two plates on the right), a control NID2695 strain expressing the *uidA* gene, but not expressing *mRFP*, was positioned for comparison. Plates were imaged at white light (VL) and in a setup detecting red fluorescence (FL). White colonies which also has red fluorescence signal represent the successful replacement of *uidA* by *mRFP*-GEC. Blue colonies with no fluorescence signal represent the colonies in which *mRFP*-GEC was not integrated into the *uidA* locus. Blue colonies with fluorescence signal represent heterokaryons.

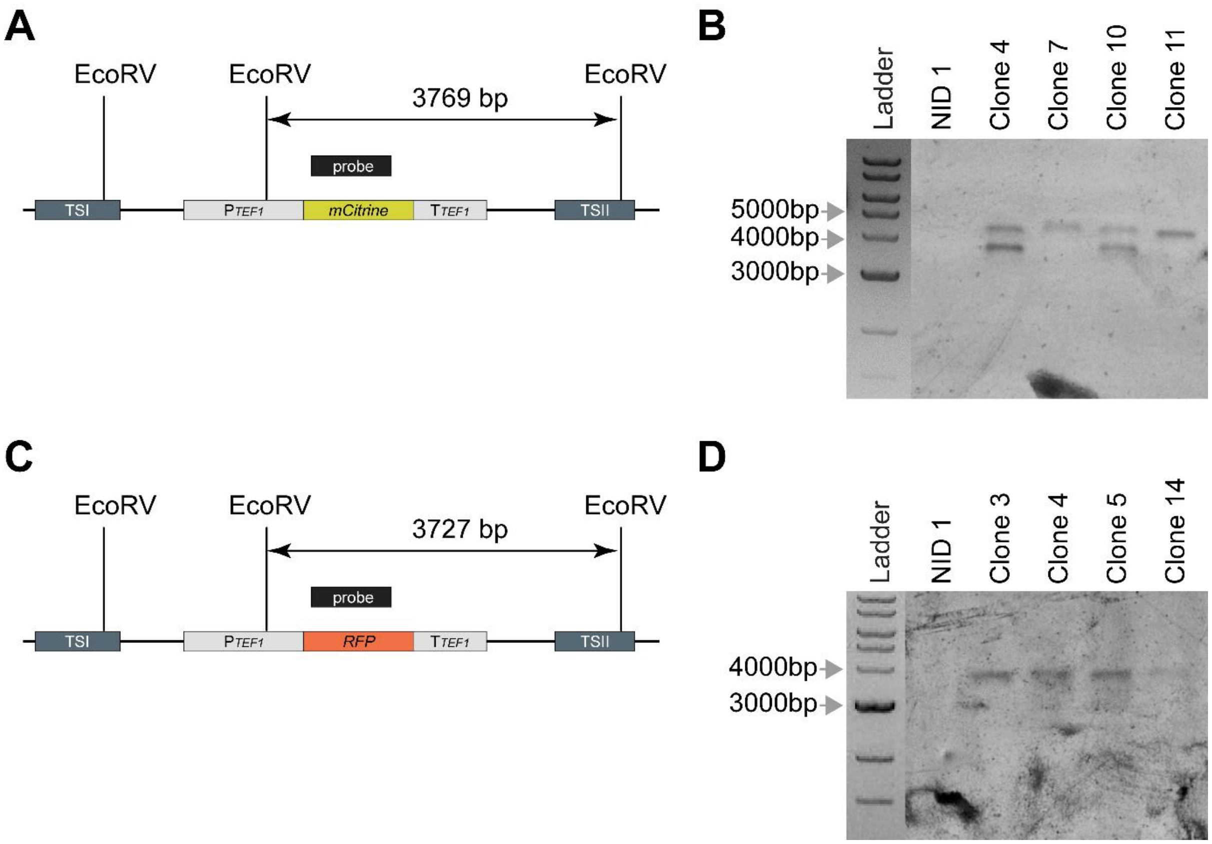

Supplementary Figure S8 Southern blots of GEC insertion strains generated by *in vivo* assembly. **(A)** Schematic representation of the *mCitrine* gene inserted into COSI-1. Positions of the EcoRV cut sites and of the detection probe are indicated. **(B)** A reference NID1 strain and four random colonies were analyzed for integration of the *mCitrine* gene into COSI-1. Genomic DNA was purified and digested with EcoRV and then analyzed by Southern blotting. **(C)** Schematic representation of the *mRFP* gene inserted into COSI-1. Positions of the EcoRV cut sites and of the detection probe are indicated. **(D)** A reference NID1 strain and four random colonies were analyzed for integration of the *mRFP* gene into COSI-1. Genomic DNA was purified and digested with EcoRV and then analyzed by Southern blotting.

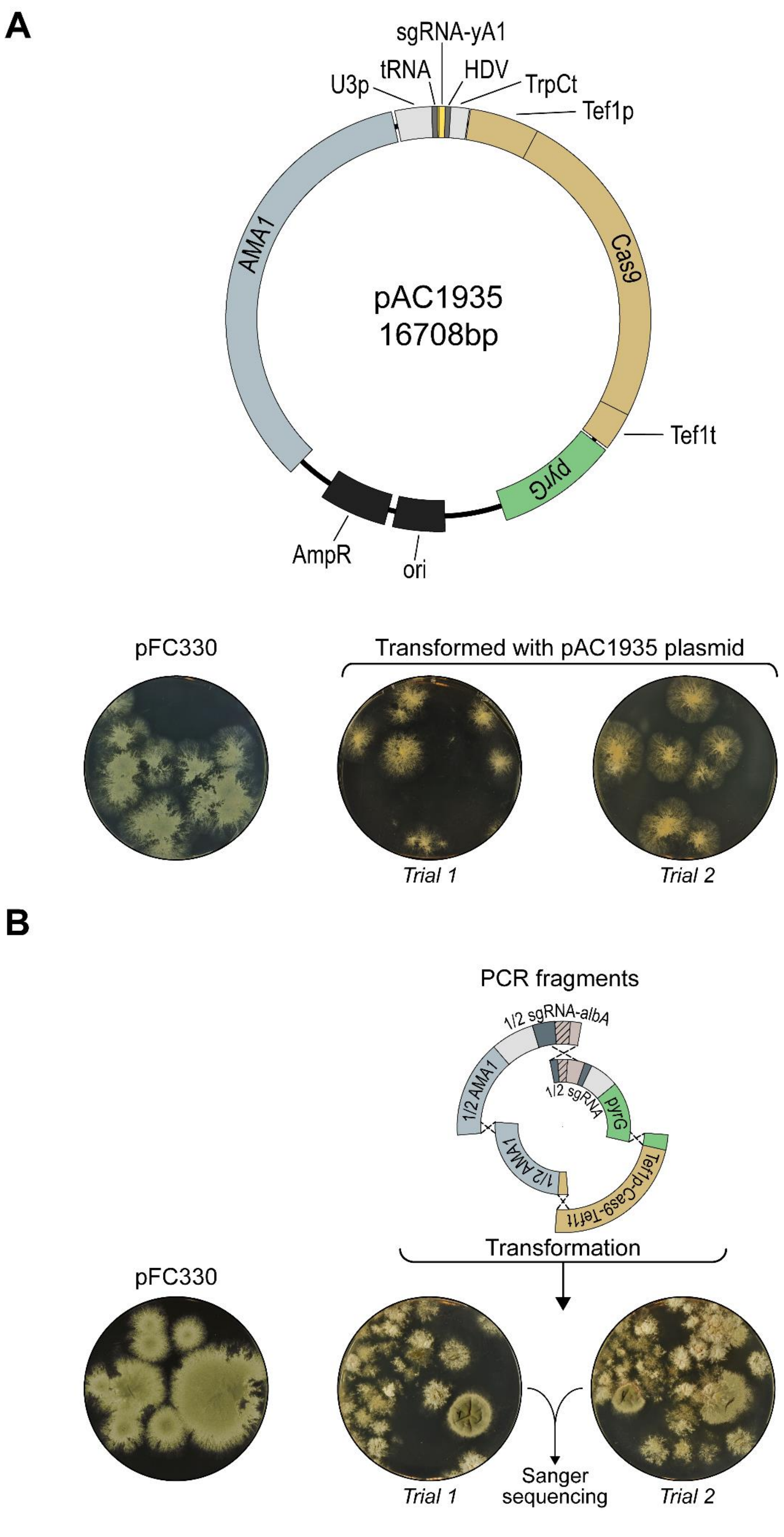
A
sgRNA-yA1
tRNA
HDV
U3p
TrpCt
Tef1p
AMA1
Cas9
pAC1935
16708bp
Tef1t
pyrG
AmpR
ori
pFC330
Transformed with pAC1935 plasmid
Trial 1
Trial 2
B
PCR fragments
1/2 sgRNA-albA
1/2 AMA1
1/2 sgRNA
pyrG
1/2 AMA1
Tef1p-Cas9-Tef1t
Transformation
pFC330
Trial 1
Sanger sequencing
Trial 2

Supplementary Figure S9 Validation of the tRNA::sgRNA::HDV setup for sgRNA release. **(A)** To demonstrate functionality of the tRNA::sgRNA::HDV cassette, we transformed NID1 strain with pFC330, an empty CRISPR vector (left) and with pAC1935 (*tRNA::yA1-sgRNA::HDV* vector) along with the repair oligonucleotide GE-Oligo1 (right). pAC1935 produces Cas9 and an efficient sgRNA targeting *yA*. The resulting DNA DSB in *yA* can be repaired by GW-Oligo1. If so, a non-sense mutation is introduced into *yA* changing conidia color from green to yellow, for details concerning the sgRNA and GW-Oligo1, see (2). Based on the two experiments, we judge the mutation efficiency is > 90%. **(B)** Construction of a *tRNA::albA-sgRNA::HDV* ESF-CRISPR vector in NID1 strains by *in vivo* assembly. NID1 was transformed with an empty CRISPR plasmid pFC330 (left) or the four different PCR fragments required for *albA* ESF-CRISPR plasmid assembly (right). Note the *albA-sgRNA* does not have a target sequence in the *A. nidulans* genome, but rather matches *albA* in *A. aculeatus*. Hence, it does not introduce toxic DNA DSBs in *A. nidulans* and the number of transformants therefore reflect the efficiency of the assembly process. Three transformants were randomly selected for sequence analysis. In all three cases, the sgRNA expression cassette was correctly assembled.

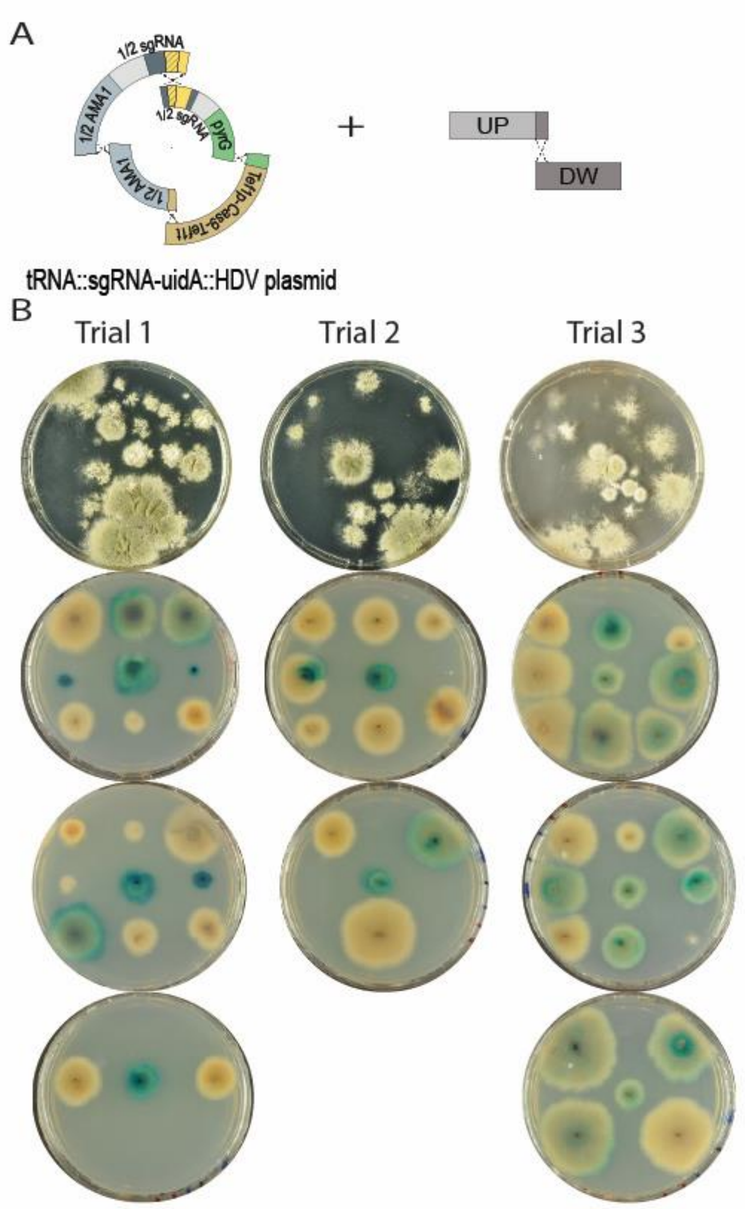

Supplementary Figure S10 Cloning- and marker-free gene deletion by *in vivo* assembly. **(A)** *in vivo* assembly of the ESF-CRISPR plasmid, and in parallel, *in vivo* assembly of a gene-targeting substrate for marker-free deletion of *uidA* in COSI-1 (1). **(B)** All PCR fragments shown in (A) were co-transformed, in three trials, into NID2695. All transformants from each trial were transferred to solid MM media containing X-Gluc. In the middle of each plate, a control strain NID2695 expressing the *uidA* gene was positioned for comparison. White colonies indicate a successful marker-free deletion of the *uidA* gene in COSI-1 using the gene-targeting substrate assembled by *in vivo* fusion of two PCR fragments for repair.

Supplementary Table S1 Vectors used in this study.

Plasmid ID	Description[a]	Purpose	Reference
pAC1767	*An_argB*-AMA1::*uidA-5tr*	For gap repair assay	This study
pAC1688	*An_argB*-AMA1::*uidA*	Control vector for gap repair experiments	This study
pDIV083	UP(*An*)-A-*An*_P*gpdA*-*uidA*-*An*_T*trpC*-B-DW(*An*)	Vector for amplification of P*gpdA*-*uidA*-T*trpC*	(1)
pAC573	*An_argB*-AMA1::PacI/Nt.BbvCI	Vector for constructing pAC1688 and control vector for AMA1 assembly	(2)
pAC572	*Af_pyrG* -AMA1::PacI/Nt.BbvCI	Control vector for AMA1 assembly and gene deletions	(2)
pDIV088	A-*An*_P*gpdA*-*mCitrine*-*An*_T*trpC*-B	Template for Southern blot probe	
pDIV089	A-*An*_P*gpdA*-*mRFP*-*An*_T*trpC*-B	Template for Southern blot probe	(1)
pFC330	*Af_pyrG*-AMA1::PacI/Nt.BbvCI-*cas9*	Control vector for GEC integrations	(3)
pDIV073	pFC330*::uidA-gRNA2*	Cas9 vector for GEC integrations	(1)
pAC1935	*cas9-Af_pyrG*-AMA1::*Af_U3p-tRNA-yA-sgRNA1-HDV-An_TtrpC*	Cas9 vector for *yA1* targeting	This study

[a]An – *Aspergillus nidulans*; Af – *Aspergillus fumigatus*.

Supplementary Table S2 Strains used in this study.

Strain ID[a]	Genotype	Purpose	Reference
NID1	*veA1*, *argB2*, *pyrG89*, *nkuAΔ*	Gap repair assays; gene deletions	(4)
NID5	*veA1*, *argB2*, *pyrG89*	Gap repair assays	IBT 27263
NID2695	*veA1*, *argB2*, *pyrG89*, *nkuAΔ*, IS1::A-P*gpdA*-*uidA*-T*trpC*-B	AMA1 assembly; GEC integration	(1)
ACU59	*pyrG1*, *akuAΔ*, IS1::A-P*gpdA*-*uidA*-T*trpC*-B	AMA1 assembly; GEC integration	(1)
NIG158	*pyrG1*, *kusAΔ*, IS1::A-P*gpdA*-*uidA*-T*trpC*-B	AMA1 assembly; GEC integration	(1)
ORY7	*pyrGΔ*, *ku70Δ*, IS1::A-P*gpdA*-*uidA*-T*trpC*-B	AMA1 assembly; GEC integration	(1)

[a] NID – *Aspergillus nidulans*; ACU – *Aspergillus aculeatus*; NIG – *Aspergillus niger*; ORY – *Aspergillus oryzae*.

Supplementary Table S3 List of primers used in this study.

Primer ID	Sequence[a] 5' → 3'	Purpose[b]
**Primers for fragment amplification with increasing ends-out homologies in gap repair assay**		
ZJ142	GGGTTTAAUATTCCCTTGTATCTCTACACACAGG	To amplify a USER cloning compatible *uidA* reporter gene cassette
ZJ428	GGTCTTAAUGGGCGCTTACACAGTACACG	To amplify a USER cloning compatible *uidA* reporter gene cassette
C407	GCCATTAACCTAGGTACAGAAGTC	To construct 25 bp homology overhang fragment
C408	TGATCGTTAAAACTGCCTGGC	To construct 25 bp homology overhang fragment
C409	GTGTATAGCCGTCGGCGAAATAG	To construct 50 bp homology overhang fragment
C410	ATTACGAATATCTGCATCGGCGAAC	To construct 50 bp homology overhang fragment
C411	GAGCTTCATCGAATCACCGG	To construct 100 bp homology overhang fragment
C412	CAACCTTTCGGTATAAAGACTTCGC	To construct 100 bp homology overhang fragment
C413	GATGATTATTAATCCGGGACCGG	To construct 200 bp homology overhang fragment
C414	CGTATAGCCGCCCTGATG	To construct 200 bp homology overhang fragment
C415	CTGGAGTGACCCAGAGGG	To construct 400 bp homology overhang fragment
C416	GTGGTGTAGAGCATTACGCTG	To construct 400 bp homology overhang fragment
C417	TTAAATCAGGAGATATAGCATGATCTCTAG	To construct 800 bp homology overhang fragment
C418	CACCATCAGCACGTTATCGAATC	To construct 800 bp homology overhang fragment
C419	TTTCGACACTGAAATACGTCGAG	To construct 1600 bp homology overhang fragment
C420	GACTTCGGTTTGCGGTCG	To construct 1600 bp homology overhang fragment
**Primers for AMA1 plasmid assembly**		
PR_DIV1387	GAACCAGAAAGGCAAGGCAG	Amplification of I half of AMA1
PR_DIV1390	TGAGGTCTTAATTAAACCCTCAGC	Amplification of I half of AMA1
PR_DIV1395	AAGATCTCATGGTCATAGCTGTTTCCGCTGAGGGTTTAATTAAGACCTCAATTCCCTTGTATCTCTACACACAGG	Amplification of ANP*gpdA* with 50 bp overhang
PR_DIV1396	GCGGTAGTGATGTCTGCTCAA	Amplification of ANP*gpdA*
PR_DIV1397	CATCGCAGCTTGACTAACAGCTACCCCGCTTGAGCAGACATCACTACCGCATGGTGAGCAAGGGCGAG	Amplification of *mCitrine* with 50 bp overhang
PR_DIV1398	TATCCAGATCGTCAAGCTGTTTGATGATTTCAGTAACGTTAAGTGGATCCTTACTTGTACAGCTCGTCCATGC	Amplification of *mCitrine* with 50 bp overhang
PR_DIV1399	GGATCCACTTAACGTTACTGAAATC	Amplification of ANT*trpC*
PR_DIV1400	CTGCGTCCGGGTCTGTTTCATTAGATGACTACCCCAAAAAACCGCGGAATGGGCGCTTACACAGTACACG	Amplification of ANT*trpC* with 50 bp overhang
PR_DIV1401	ATTCCGCGGTTTTTTGGG	Amplification of AN P-*argB*-T
PR_DIV1402	TACGCGTTAAGCTTGGCACTGGCCGTCAGCTGCTTCGTCGATTAAACCCTACCTACAGCCATTGCGAAACC	Amplification of AN P-*argB*-T with 50 bp overhang
PR_DIV1389	AGGGTTTAATCGACGAAGCAG	Amplification of II half of AMA1
PR_DIV1388	ATCACTTCCAACACTCTTGCTGA	Amplification of II half of AMA1
PR_DIV1391	AAGATCTCATGGTCATAGCTGTTTCCGCTGAGGGTTTAATTAAGACCTCACGAGACAGCAGAATCACCGC	Amplification of ANP*TEF1* with 50 bp overhang

PR_DIV1390	TGAGGTCTTAATTAAACCCTCAGC	Amplification of ANP*TEF1*
PR_DIV1348	CTGAGCACTTCTCCCTTTTATATTCCACAAAACATAACACAACCTTCACCATGGCCTCCTCCGAGGAC	Amplification of *mRFP* with 50 bp overhang
PR_DIV1349	TCGTAAAGGCTTTTTTAAGGAAGTCATAACGGCATAAATCGAATGTCCGCTTAGGCGCCGGTGGAGT	Amplification of *mRFP* with 50 bp overhang
PR_DIV430	GCGGACATTCGATTTATGCC	Amplification of ANT*TEF1*
PR_DIV1392	CCCCGGCCGGGTACCGAGCTCGAATTCGTAATCATGTCATATTACCTAGTGTATTGGGATGAATTTTGTATGCAC	Amplification of ANT*TEF1* with 50 bp overhang
PR_DIV1393	ACTAGGTAATATGACATGATTACGAATTC	Amplification of AF P-*pyrG*-T
PR_DIV1394	TACGCGTTAAGCTTGGCACTGGCCGTCAGCTGCTTCGTCGATTAAACCCTAGTGGGGATGCCTCAATTGT	Amplification of AF P-*pyrG*-T with 50 bp overhang
**Primers for gene integrations**		
PR_DIV432	AGGTGTAAAAGTAGGGAGCGGTAG	Amplification of A
PR_DIV1344	TCACTGTCCGCTTGCAGAGT	Amplification of A
PR_DIV1346	TTGGTGGGTAAAGTCTGGCAGGTACCGTCCACTCTGCAAGCGGACAGTGACGAGACAGCAGAATCACCGC	Amplification of ANP*TEF1* with 50 bp overhang
PR_DIV427	GGTGAAGGTTGTGTTATGTTTTGTG	Amplification of ANP*TEF1*
PR_DIV1350	CTGAGCACTTCTCCCTTTTATATTCCACAAAACATAACACAACCTTCACCATGGTGAGCAAGGGCGAG	Amplification of *mCitrine* with 50 bp overhang
PR_DIV1351	TCGTAAAGGCTTTTTTAAGGAAGTCATAACGGCATAAATCGAATGTCCGCTTACTTGTACAGCTCGTCCATGC	Amplification of *mCitrine* with 50 bp overhang
PR_DIV430	GCGGACATTCGATTTATGCC	Amplification of ANT*TEF1*
PR_DIV1347	GGGAGCTAGGTATTCCCTTCGGTAGAAGTAGCAGGAGTAGCGTACATAGGGTATTGGGATGAATTTTGTATGCAC	Amplification of ANT*TEF1* with 50 bp overhang
PR_DIV1345	CCTATGTACGCTACTCCTGCTACT	Amplification of B
PR_DIV435	GAGGAGAGTGGATGGATAGTCTGG	Amplification of B
PR_DIV1348	CTGAGCACTTCTCCCTTTTATATTCCACAAAACATAACACAACCTTCACCATGGCCTCCTCCGAGGAC	Amplification of *mRFP* with 50 bp overhang
PR_DIV1349	TCGTAAAGGCTTTTTTAAGGAAGTCATAACGGCATAAATCGAATGTCCGCTTAGGCGCCGGTGGAGT	Amplification of *mRFP* with 50 bp overhang
**Primers for gene deletions**		
C606	CGTGGAGTTACCAGTGATTG	Amplification of AF P-*pyrG*-T
C607	CTAGATGACTGGTAGGAATCTAAC	Amplification of AF P-*pyrG*-T
C608	CGGACCTGACAATTCTCGACG	Amplification of AN*yA*Δ TSI
C610	AGGCAGAACTGTAGAAGATAAAACATTGGTCAATCACTGGTAACTCCACGGCTGGATCCCGGAGGAATC	Amplification of AN*yA*Δ TSI with 50 bp overhang
C612	CCCCCAATCGTCAAGGTTTTCCCTTTGTTAGATTCCTACCAGTCATCTAGGGTGAGCTCTCATATTCGTACTTAC	Amplification of AN*yA*Δ TSII with 50 bp overhang
C613	CCGGTCAAAACTGCATCGG	Amplification of AN*yA*Δ TSII
C614	CTCTGGAACAGTCTCGCC	Amplification of AN*wA*Δ TSI
C616	AGGCAGAACTGTAGAAGATAAAACATTGGTCAATCACTGGTAACTCCACGGATCAGGAGAAGGAGAGTCAAG	Amplification of AN*wA*Δ TSI with 50 bp overhang
C618	CCCCCAATCGTCAAGGTTTTCCCTTTGTTAGATTCCTACCAGTCATCTAGGGCAAAGGAGCTTGCTACTTTC	Amplification of AN*wA*Δ TSII with 50 bp overhang
C619	AAGCCGACACCTGACTCG	Amplification of AN*wA*Δ TSII
**For construction of pAC1935**		
oKST_83	ACCCCAAUGGAAACGGTGAGAGTCCA	AMA FW
oKST_289	AAGTTGAUAACGGACTAGCCTTATTTTAAC	traRNA sgRNA backbone RV primer
oKST_288	ATCAACTUGAAAAAGTGGCACCGAGTC	traRNA sgRNA backbone FW primer
oKST_230	ACTGTTCTUCTAGTGTAGCCGTAGTTAGC	int Ori USER RV
oKST_229	AAGAACAGUATTTGGTATCTGCGCTCTG	int Ori USER FW
oKST_82	ATTGGGGUACTAACATAGCCATCAAATGC	AMA RV
**ESF-CRISPR albA test vector bio-blocks**		

oKST_335	CAGTCATCTAGCAAGGCGGCCGCAGCTAGCACAATTGAGGCATCCCCACTCGAGACAG CAGAATCACCGC	FW ANT*TEF1* with 50 bp HR to AF P-*pyrG*-T
oKST_337	TACGCGTTAAGCTTGGCACTGGCCGTCAGCTGCTTCGTCGATTAAACCCTGTATTGGGA TGAATTTTGTATGCAC	RV ANT*TEF1* 50 bp HR AMA part 1
PR_DIV1389	AGGGTTTAATCGACGAAGCAG	Amplification of II half of AMA1
PR_DIV1388	ATCACTTCCAACACTCTTGCTGA	Amplification of II half of AMA1
PR_DIV1387	GAACCAGAAAGGCAAGGCAG	Amplification of I half of AMA1
oKST_330	GCTATTTCTAGCTCTAAAACCGATTCTTCAACATGTCGCCTGCATCATCCGTGAATCGAA	60 bp overhang with ACU *albA* sgRNA RV
oKST_329	TTCGATTCACGGATGATGCAGGCGACATGTTGAAGAATCGGTTTTAGAGCTAGAAATA GC	60 bp overhang ACU *albA* sgRNA FW
oKST_336	AGTGGGGATGCCTCAATTGT	P-*pyrG*-T Rv
uidA* deletion, ESF-CRISPR vector expressing an sgRNA targeting *uidA		
oKST_356	TTCGATTCACGGATGATGCACGCAGGTGATCGGACGCGTCGTTTTAGAGCTAGAAATA GC	60 bp overhang with *uidA* sgRNA FW
oKST_357	GCTATTTCTAGCTCTAAAACGACGCGTCCGATCACCTGCGTGCATCATCCGTGAATCGAA	60 bp overhang with *uidA* sgRNA RV
oKST_358	GGTCGCGGAGTTCATAATCG	FW IS1-A repair
oKST_359	TCACTGTCCGCTTGCAGAG	RV IS1-A repair
oKST_360	TTGGTGGGTAAAGTCTGGCAGGTACCGTCCACTCTGCAAGCGGACAGTGATCCTATGTA CGCTACTCCTG	FW B-IS1 repair with 50 bp overlap to A
oKST_361	TAGGATTGAGATTGCAATACCC	RV B-IS1 repair
**Primers for diagnostic PCR and sequencing**		
C413	GATGATTATTAATCCGGGACCGG	Amplification & sequencing of blue gap repair colonies
C414	CGTATAGCCGCCCTGATG	Amplification & sequencing of blue gap repair colonies
PR_DIV1745	GCTTGAGAGGGCACGTATGG	Amplification of AMA1 plasmids
CSN37	TTTATCTGTTGTGCCACCAGTCTC	Amplification of *yAΔ* locus
M91	GTAACCTCTGTCGCATCTG	Amplification of *yAΔ* locus
PR_DIV1751	CCAGATGATGGCTCAGGCA	Amplification of *wAΔ* locus
M87	CACTCTGGAAACGAACTC	Amplification of *wAΔ* locus
J43	GGACAACGGGAAGAGGCTCAG	Amplification of mCitrine or RFP in AN *uidA* locus
J44	GGAGAGGGAGAGAAGAAGAAGGG	Amplification of mCitrine or RFP in AN *uidA* locus
PR_DIV341	GCATTCCAAGTATTGAACCTTCTC	Amplification of RFP in ACU *uidA* locus
PR_DIV342	GATGTCTCGCACGAGTCCAGT	Amplification of RFP in ACU *uidA* locus
ZJ79	GTTCCACGGGAGGTGTGAATC	Amplification of RFP in ASN *uidA* locus
ZJ96	CTGTGTGACTGGTAGGTTCTGAGTG	Amplification of RFP in ASN *uidA* locus
PR_DIV1127	GCAGTTTGGGTGTGTGGTGTT	Amplification of RFP in AO *uidA* locus
PR_DIV1128	GAGTGGGTTAATGTTGCGTCG	Amplification of RFP in AO *uidA* locus
EDR08	CAAGTCAGCCAACTGCAAACAG	Sequencing of AMA1-mCitrine plasmids
CSN46	TGCGTCAGTCCAACATTTGT	Sequencing of AMA1-mCitrine plasmids
PR_DIV1748	GCATCAAGGTGAACTTCAAGATC	Sequencing of AMA1-mCitrine plasmids
CSN159	CCTATGAGTCGTTTACCCAG	Sequencing of AMA1-mCitrine plasmids
ZJ301	GGTTTCGTTGTCAATAAGGGAA	Sequencing of AMA1-mCitrine plasmids

oKST_72	TGCTCGGAATCCATATTG	Sequencing of AMA1-RFP plasmids and integration of mCitrine/RFP-GEC
CSN364	CTTCTCTGCTCAGCACCTCTACG	Sequencing of AMA1-RFP plasmids and integration of mCitrine/RFP-GEC
PR_DIV482	CCAATGCGCGATTCAAGAC	Sequencing of AMA1-RFP plasmids and integration of mCitrine/RFP-GEC
CSN42	TGTAGTGCCAGTACGAGTGTTGTG	Sequencing of AMA1-RFP plasmids
CSN451	CGACAACCAAGGGAAGTCAACA	Sequencing of of *yAΔ* locus
A262	GGCTGGGAGGGTCAGAGG	Sequencing of of *yAΔ* locus
CSN592	CTTGCCGATGCTACATTGCCT	Sequencing of of *wAΔ* locus
PR_DIV1752	GCTGAACACTTAGATGCAGTCTCTC	Sequencing of of *wAΔ* locus
MF233	AGGTTCTCCTAACGCTTGGC	Sequencing of AF*PU3*
oKST_110	TGATCCACAGCGTCAAGA	Sequencing of ANP*TEF1*
oKST_352	CCCGAAACGCGTTTTATTCTTG	Sequencing ANT*TRPC*
oKST_358	GGTCGCGGAGTTCATAATCG	FW IS1-A repair
oKST_361	TAGGATTGAGATTGCAATACCC	RV B-IS1 repair
**Primers for Southern blot probe design**		
PR_DIV1428	CGTCATCAAGGAGTTCATGC	mRFP1_probe-F
PR_DIV1429	CTCGTTGTGGGAGGTGATGT	mRFP1_probe-R
PR_DIV0226	GTAAACGGCCACAAGTTCAGC	mCitrine_probe-F
PR_DIV0227	GTAGTGGTTGTCGGGCAGC	mCitrine_probe-R

[a] Color code: annealing sequence, overhangs, protospacer sequence, thymine-> uracil substitution.
[b] AN – *Aspergillus nidulans*; AF – *Aspergillus fumigatus*; TS – targeting sequence; ACU – *Aspergillus aculeatus*; ASN – *Aspergillus niger*; AO – *Aspergillus oryzae*.

REFERENCES

1. Jarczynska,Z.D., Rendsvig,J.K.H., Pagels,N., Viana,V.R., Nødvig,C.S., Kirchner,F.H., Strucko,T., Nielsen,M.L. and Mortensen,U.H. (2021) DIVERSIFY: A fungal multispecies gene expression platform. *ACS Synth. Biol.*, **10**, 579–588.

2. Nødvig,C.S., Hoof,J.B., Kogle,M.E., Jarczynska,Z.D., Lehmbeck,J., Klitgaard,D.K. and Mortensen,U.H. (2018) Efficient oligo nucleotide mediated CRISPR-Cas9 gene editing in Aspergilli. *Fungal Genet. Biol.*, **115**, 78–89.

3. Nødvig,C.S., Nielsen,J.B., Kogle,M.E. and Mortensen,U.H. (2015) A CRISPR-Cas9 system for genetic engineering of filamentous fungi. *PLoS One*, **10**, 1–18.

4. Nielsen,J.B., Nielsen,M.L. and Mortensen,U.H. (2008) Transient disruption of non-homologous end-joining facilitates targeted genome manipulations in the filamentous fungus Aspergillus nidulans. *Fungal Genet. Biol.*, **45**, 165–170.
